# Shear Conditioning Promotes Microvascular Endothelial Barrier Resilience in a Human BBB‐on‐a‐Chip Model of Systemic Inflammation Leading to Astrogliosis

**DOI:** 10.1002/advs.202508271

**Published:** 2025-08-27

**Authors:** Kaihua Chen, Isabelle M. Linares, Michelle A. Trempel, Alexis M. Feidler, Dinindu De Silva, Sami Farajollahi, Jordan Jones, Julia Kuebel, Pelin Kasap, Britta Engelhardt, Jonathan Flax, Vinay V. Abhyankar, Richard E. Waugh, Harris A. Gelbard, Niccolo Terrando, James L. McGrath

**Affiliations:** ^1^ Department of Biomedical Engineering University of Rochester Rochester NY 14627 USA; ^2^ Center for Musculoskeletal Research University of Rochester Medical Center Rochester NY 14642 USA; ^3^ Department of Neuroscience University of Rochester Rochester NY 14642 USA; ^4^ Department of Biomedical Engineering Rochester Institute of Technology Rochester NY 14623 USA; ^5^ Theodor Kocher Institute University of Bern Bern 3012 Switzerland; ^6^ Department of Urology University of Rochester Medical Center Rochester NY 14642 USA; ^7^ Center for Neurotherapeutics Discovery Department of Neurology University of Rochester Medical Center Rochester NY 14642 USA; ^8^ Department of Pediatrics University of Rochester Medical Center Rochester NY 14642 USA; ^9^ Department of Microbiology and Immunology University of Rochester Medical Center Rochester NY 14642 USA; ^10^ Department of Anesthesiology Center for Translational Pain Medicine Duke University Medical Center Durham NC 27710 USA; ^11^ Department of Cell Biology Duke University Medical Center Durham NC 27710 USA; ^12^ Department of Immunology Duke University Medical Center Durham NC 27710 USA

**Keywords:** astrogliosis, barrier resilience, fluid shear stress, human BBB‐on‐a‐chip

## Abstract

The blood–brain barrier (BBB) maintains cerebral homeostasis and protects the central nervous system (CNS) during systemic inflammation. Advanced in vitro models integrating circulation, a functional BBB, and reactive glial cells are essential for studying the link between peripheral inflammation and neuroinflammation. Fluid shear stress, a key hemodynamic parameter, strengthens microvascular barriers. This study examines endothelial shear conditioning on barrier function in a fluidic µSiM‐BBB (Microphysiological System featuring a Silicon Membrane –BBB). hiPSC‐derived brain microvascular endothelial cell monocultures are conditioned with 0.5 Pa shear stress for 48 h. Shear conditioning lowers baseline permeability, increases glycocalyx production, and reduces responses to inflammatory challenges, including barrier breakdown, ICAM‐1 upregulation, and neutrophil transmigration. Shear conditioning produces a resilient barrier function against a low‐dose inflammatory challenge (10 pg mL^−1^ TNF‐α/IL1‐β/INF‐γ) but a high‐dose challenge (50 pg mL^−1^) disrupts the barrier. Adding astrocytes as neuroinflammatory “sensors” reveals that a high‐dose inflammatory challenge activates astrocytes but only in combination with fibrinogen—a plasma protein known to trigger astrogliosis in multiple neurological conditions. This study highlights the utility of fluidic‐enabled µSiM‐BBB for investigating acute peripheral inflammation and brain injury relationships, serving as a foundation for more advanced models, including more cells of the neurovascular unit and brain parenchyma.

## Graphical abstract

Physiological flow (0.5 Pa) conditioning promotes resilience of an iPSC‐derived blood–brain barrier endothelium to mild systemic inflammation. In contrast, high “cytokine storm” levels induce barrier breakdown, fibrinogen leakage, and astrocyte activation, reconstituting the two‐hit injury mechanism on‐a‐chip. The fluidic µSiM‐BBB recapitulates peripheral‐to‐central inflammation transduction underlying brain injury in sepsis, trauma, and certain immunotherapies.

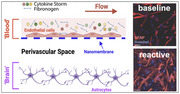

## 1 Introduction

The blood–brain barrier (BBB) is a highly specialized and dynamic interface that regulates cerebral homeostasis, selectively controlling nutrient transport while restricting the entry of pathogens and inflammatory mediators.^[^ ^1^ ^]^ In older adults and those with pre‐existing neurodegenerative disease, major insults such as infection, trauma, or surgery can lead to severe systemic inflammation, delirium, and long‐term cognitive impairment.^[^ ^2^, ^3^ ^]^ The exact molecular mechanisms that make some individuals susceptible to inflammatory insults while others are resilient are elusive. However, there is a growing recognition that BBB breakdown and leukocyte infiltration into the central nervous system (CNS) are central steps in the cascade that leads to neuroinflammation and brain injury.^[^ ^4^, ^5^, ^6^ ^]^ Complex in vitro models that employ human cells and microfluidics to simulate the peripheral circulation, the BBB, and cells of the brain parenchyma are essential, reductionist tools needed to build a mechanistic understanding of brain injury as an outcome of systemic inflammation. Such tools are also necessary for testing hypotheses regarding the differences between the “vulnerable” and “resilient” BBB, and for identifying drugs that can prevent or reverse BBB breakdown in response to peripheral inflammation.

The first key step in the development of BBB models for the study of brain injury as an outcome of peripheral inflammation is establishing a barrier that recapitulates baseline barrier function and reliably responds to inflammatory stimuli in a physiological range. Our laboratory has used iPSC‐derived brain microvascular endothelial‐like cells (BMECs) differentiated according to the extended endothelial culture method (EECM‐BMECs)^[^ ^7^ ^]^ on the µSiM (microphysiological system enabled by a silicon nanomembrane) tissue‐on‐a‐chip platform, to create a monoculture in vitro model of the BBB.^[^ ^8^ ^]^ The µSiM features ultrathin (100 nm thick), highly permeable, and optically clear membranes that are ideal for barrier modeling. Despite its simplicity, the µSiM‐BBB has been validated by demonstrating that when cultured in the µSiM, EECM‐BMECs display: 1) a requisite set of barrier‐forming junctional proteins; 2) low baseline permeability to small tracer molecules; 3) support of leukocyte transmigration, and 4) barrier breakdown in response to an inflammatory cocktail (“cytomix” = TNF‐α/ IL‐1β/ IFN‐γ) at concentrations seen in sepsis‐associated encephalitis.^[^ ^9^, ^10^ ^]^ However, all prior studies on the µSiM‐BBB have been conducted under static culture conditions, which do not fully address the multifaceted influence of blood flow on the BBB. Not only do peripheral insults and immune cells reach the BBB via blood flow, but hemodynamic shear stress is also known to have a profound impact on endothelial cell (EC) physiology.

Beginning with the original observation of EC alignment in the direction of flow 40 years ago,^[^ ^11^ ^]^ flow conditioning has been established to elicit a number of beneficial physiological changes to the cell phenotype including, improved barrier function,^[^ ^12^ ^]^ enhanced expression of anti‐inflammatory genes (e.g., eNOS, KLF2/4),^[^ ^13^, ^14^ ^]^ increased glycocalyx biosynthesis,^[^ ^4^ ^]^ and dampened inflammatory responses.^[^ ^15^, ^16^, ^17^ ^]^ Critically, in vivo leukocytes interact with ECs through rolling, arrest, and diapedesis, which are inherently flow‐dependent processes or flow‐modulated,^[^ ^12^, ^18^ ^]^ and static models overestimate adhesion compared to those with physiological shear.^[^ ^19^, ^20^ ^]^ Despite this physiological relevance, adding a fluidic circuit to an in vitro model increases experimental complexity and reduces throughput,^[^ ^21^ ^]^ and static BBB models have played a foundational role in building a mechanistic understanding of BBB functions under well‐controlled conditions.^[^ ^22^, ^23^, ^24^ ^]^ Thus, we first sought to define the impact of fluid flow for the purpose of modeling systemic inflammatory factors in the µSiM‐BBB.

Leveraging a recent addition to the modular µSiM platform—a fluidic insert that converts the static culture device to a closed microfluidic device^[^ ^13^, ^25^ ^]^—we now conduct the first flow‐based experiments in the µSiM‐BBB. We conditioned EECM‐BMECs at a wall shear stress of 0.5 Pa (5 dyne cm^−2^), a regime matching the high‐end levels of shear stress experienced in post‐capillary venules (0.1–0.6 Pa),^[^ ^26^, ^27^ ^]^ which are the most inflammation‐responsive segment of the microvasculature and the principal site of leukocyte extravasation across the BBB in vivo.^[^ ^28^, ^29^ ^]^ We show that this shear stress value conditions EECM‐BMECs within 48 h to produce a range of anticipated shear responses including cell alignment with flow, barrier enhancement, and the upregulation of glycocalyx. Most significantly, we found that pre‐exposure to shear stress results in a barrier that is more resilient to a circulating inflammatory challenge. These studies affirm the use of fluid shear preconditioning to model BBB responses to systemic inflammation.

To demonstrate the utility of the fluidic µSiM‐BBB in modeling neuroinflammatory cascades triggered by systemic inflammation, we incorporated primary human astrocytes (PHAs) in the basolateral (“brain”) compartment of the device and monitored their activation through morphological transformation (hypertrophy, process elongation) driven by glial fibrillary acidic protein (GFAP) polymerization. In this way, astrocytes function as neuroinflammatory “sensors” in the µSiM‐BBB, by displaying morphological changes similar to those observed in the brain following acute injury or as a consequence of chronic neurodegenerative diseases.^[^ ^30^, ^31^, ^32^, ^33^ ^]^ We found that pathological high‐dose cytomix (50 pg mL^−1^)—sufficient to destabilize shear‐conditioned barriers—failed to activate PHAs (primary/normal human astrocytes). This aligned with monoculture data showing astrocytes are highly resistant to cytokine‐driven activation. Therefore, we introduced fibrinogen, a serum coagulation protein known to trigger astrogliosis^[^ ^34^ ^]^ in multiple neurologic conditions, including traumatic brain injury (TBI),^[^ ^15^ ^]^ as an additional circulating factor. Our studies found that adding fibrinogen to a high dose of circulating inflammatory cytokines resulted in astrocyte activation, mirroring clinical observations in which fibrinogen leakage into the brain parenchyma triggers astrogliosis.^[^ ^35^ ^]^ Our studies establish the fluidic µSiM‐BBB as a platform for creating reductionist models to investigate the links between acute peripheral inflammation and brain injury, and to develop or screen for protective therapies.

## 2 Results

### 2.1 Development of a Modular Fluidic BBB‐on‐a‐Chip (µSiM‐BBB)

**Figure 1:**
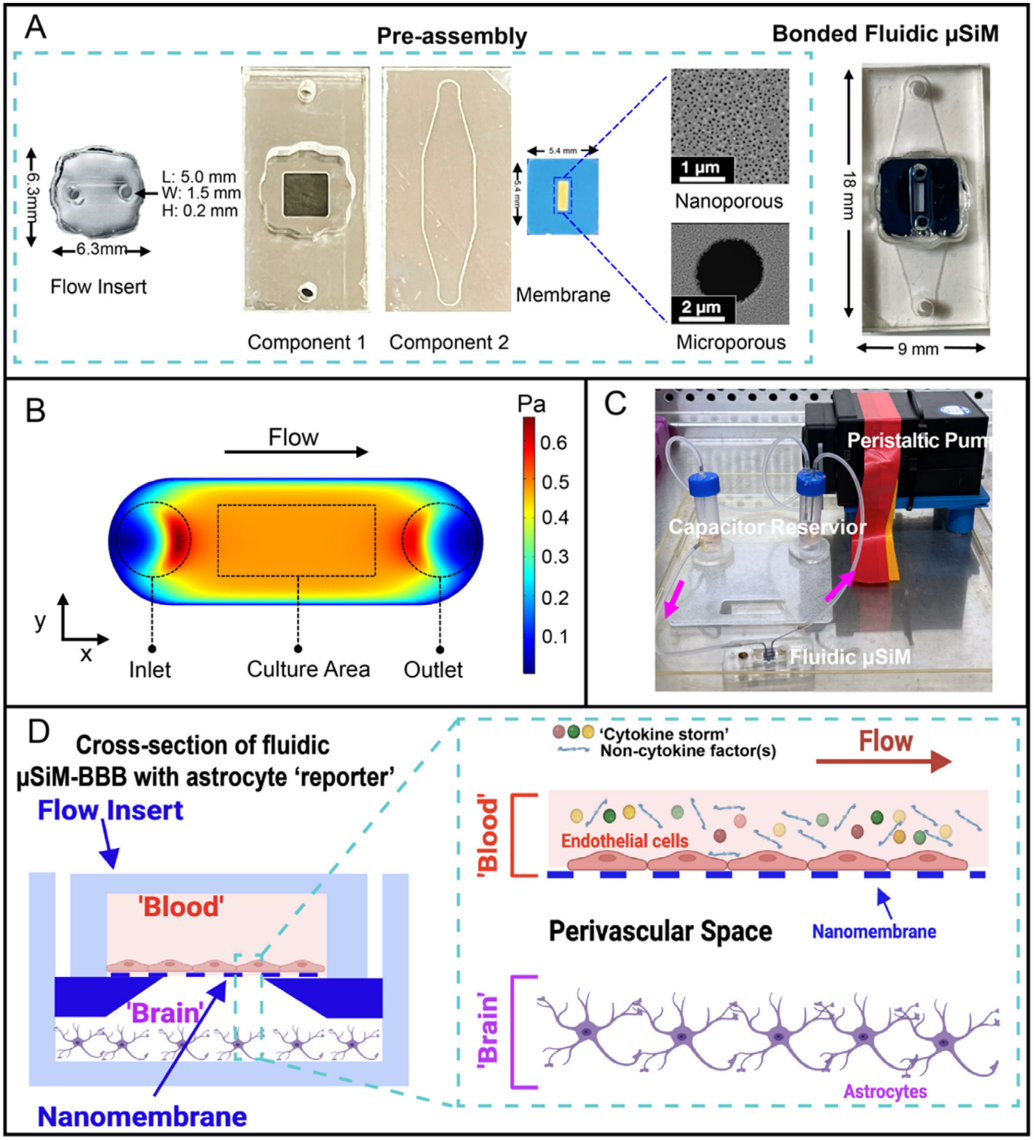
Development of fluidic µSiM‐BBB. A) *Left*: Pre‐assembled components, including the PDMS flow insert and the open‐well µSiM device components: component 1 (top well), component 2 (bottom channel), and silicon nanomembranes (nanoporous and dual‐scale). *Right*: Fully bonded fluidic µSiM device. B) COMSOL simulation depicting a steady shear stress distribution over the culture area of the membrane at 290 µL min^−1^ flow rate, validating the effective design of the device. C) Custom flow circuit used for continuous fluidic experiments, featuring a peristaltic pump for media circulation, a reservoir for cell media supply, a capacitor for damping flow fluctuations, and acrylic stages to secure the entire setup. D) Cross‐sectional schematic of the µSiM‐BBB, illustrating EECM‐BMECs cultured within the flow channel (“Blood”) and PHAs seeded on the bottom channel floor (“Brain”), with a “perivascular space (PVS)” separating the two compartments.

To investigate the role of hemodynamic regulation of barrier function under systemic inflammation, we employed a flow insert that transforms our established open‐well µSiM barrier modeling platform^[^ ^8^ ^]^ into a closed microfluidic device. This recent innovation enables the simulation of apical flow over an endothelial layer while preserving device transparency for live cell imaging of barriers and underlying tissue.^[^ ^13^, ^25^ ^]^ The base µSiM device features ultrathin, optically clear, and highly permeable nanoporous silicon nitride membranes as the culture substrate for barrier models.^[^ ^8^, ^13^, ^36^, ^37^ ^]^ The modularity of the µSiM enables a choice of membrane appropriate for different models or assays. The current work used both nanoporous membranes (100 nm thick, 60 nm pores, 15% porosity) that allow barrier development and small‐molecule exchange but not cell transmigration, and dual‐scale variants that superimpose 3 µm pores (0.625% added porosity) onto the nanoporous background to enable complete leukocyte transmigration into the bottom channel (**Figure** **1**A).

We used the flow insert in combination with a custom flow circuit (Figure 1C) comprising a pulsation‐dampening capacitor, a media reservoir, and a peristaltic pump.^[^ ^38^ ^]^ We used validated COMSOL simulations of the flow channel geometry^[^ ^13^ ^]^ to confirm that a flow rate of 290 µL min^−1^ is expected to produce a uniform 0.5 Pa (5 dynes cm^−2^) shear stress across all cells on the membrane region (Figure 1B). This shear stress value is on the upper end of the hemodynamic forces expected in the transition from microcapillaries to post‐capillary venules, where leukocyte trafficking occurs.^[^ ^26^, ^27^ ^]^ Precision control over flow allows us, for the first time, to study shear‐conditioning on iPSC‐based BMECs derived from the extended endothelial culture method.^[^ ^7^, ^39^ ^]^ EECM‐BMECs were cultured on collagen IV/fibronectin‐coated membranes under shear stress in the luminal (“blood”) compartment, while PHAs adhered to collagen I/fibronectin‐coated channel floors in the static abluminal (“brain”) compartment directly beneath the EECM‐BMEC barrier (Figure 1D). Note that the physical separation of astrocytes and EECM‐BMECs occurs in the post‐capillary venule and is prominent under inflammatory conditions^.[^ ^28^, ^29^ ^]^ Thus, the basal microfluidic channel of the µSiM serves as a 310 µm‐tall model of the perivascular space (PVS) in post‐capillaries—comparable to the sub‐2 mm PVS observed in the human brain.^[^ ^40^ ^]^ We did not include pericytes in these studies, as our prior study found they did not contribute to barrier resistance to small molecule permeation under either baseline or inflamed conditions.^[^ ^9^ ^]^ This approach allows us to concentrate on the critical interactions central to our investigation.

### 2.2 Physiologically High Shear Stress (0.5 Pa) Streamlines EECM‐BMEC Conditioning Workflows and Results in a 2‐h Post‐Flow Experimental Window

**Figure 2:**
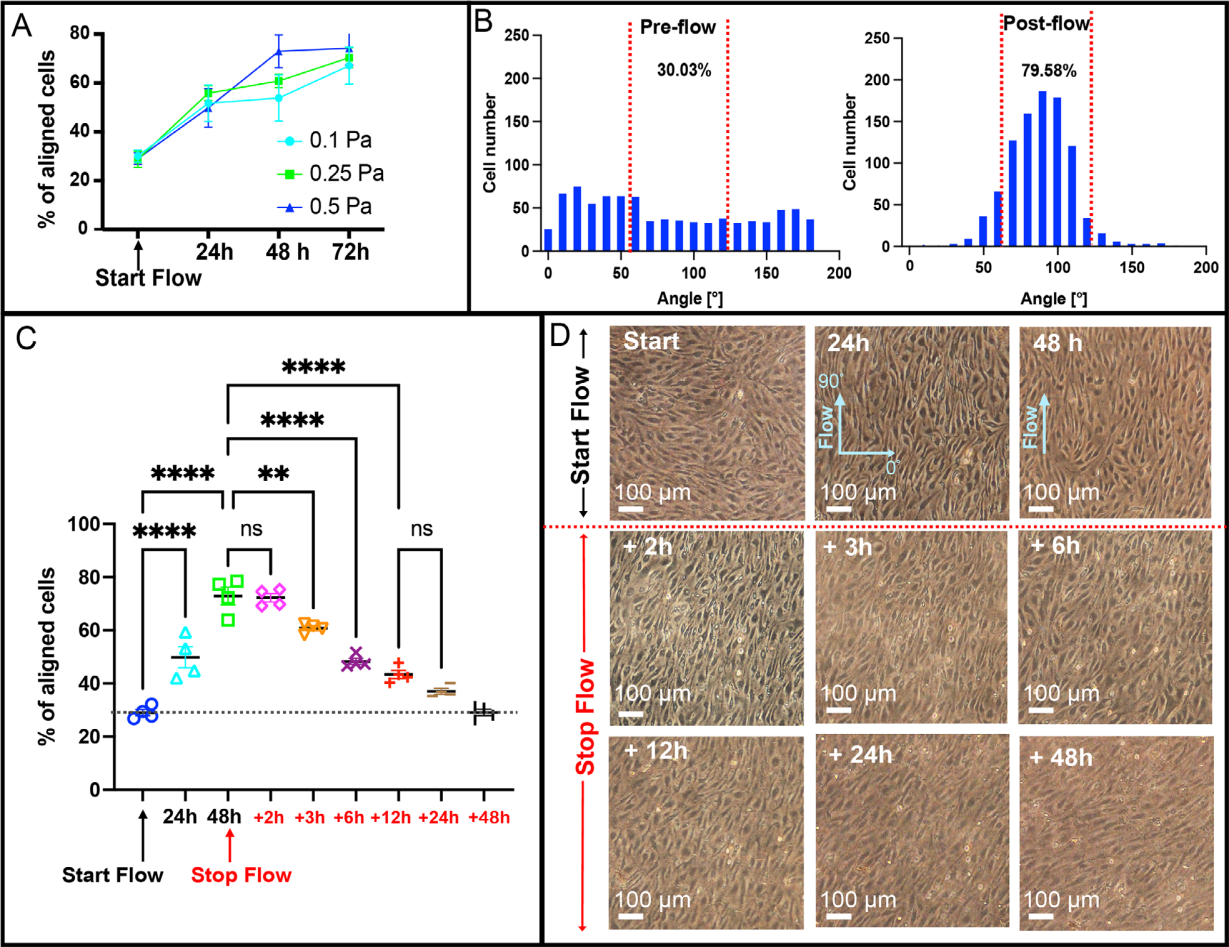
Morphological Response of EECM‐BMECs to the Onset and Cessation of Flow. A) Comparison of cell alignment (between 60° and 120°) in the flow directions (90°) under varying shear stresses (0.1, 0.25, and 0.5 Pa) over 24, 48, and 72 h. While lower shear stresses (0.1 and 0.25 Pa) required 72 h to achieve similar alignment levels, a higher shear stress of 0.5 Pa required only 48 h (Statistical comparison is shown in Figure S1, Supporting Information). n = 4–7 for each condition. Post 72 h of flow, there was no significant difference in alignment across all shear stresses, including the maintenance of alignment under 0.5 Pa between 48 and 72 h (Figure S1, Supporting Information). B) Distribution of cell orientations (0–180°, bin = 10°) relative to flow direction (90°) in the pre‐shear conditioning group compared to the post‐shear group (290 µL min^−1^, 0.5 Pa for 48 h), showing a notable enhancement of alignment after flow conditioning. C) Quantification of cell alignment percentages under a SS of 0.5 Pa for up to 48 h, with a sustained morphological change lasting for 2 h post‐flow, followed by a gradual decrease in alignment over 48 h. One‐way ANOVA test with Tukey's post‐hoc, ∗∗∗∗*p* < 0.0001, ∗∗∗*p* < 0.001, ∗∗*p* < 0.01, ∗*p* < 0.05, ns > 0.05. Data are mean ± standard deviation, n = 4. D) Phase‐contrast images showcasing cellular morphology before and after shear stress exposure at 24 and 48 h, and during the post‐flow period at 2, 6, 12, 24, 48, and 96 h. Images highlight the initial retention and subsequent decline in alignment post‐shear. Scale bar = 100 µm.

We sought to optimize workflows so that we could observe the potential benefits of shear conditioning with minimum experimental time. In these studies, we applied a range of post‐capillary venule shear stress levels (0.1, 0.25, and 0.5 Pa) to EECM‐BMEC monolayers for 72 h following 4 days of static culture. Phase‐contrast imaging and quantitative analysis (see Experimental Section, Equation 1) of cell orientation relative to the flow direction revealed that 0.5 Pa shear stress achieved conditions such that > 70% of EECM‐BMECs were orientated within 30° of the flow direction after 2 days of flow and these values did not change with an additional day of flow (**Figure** **2**A,B; Figure S1, Supporting Information). Lower values of shear stress (0.1 and 0.25 Pa) trended to the same plateau values after 72 h of flow (Figure 2A). Assuming EECM‐BMEC alignment is sufficient as a surrogate metric of flow‐induced barrier enhancement, we conclude that two days of shear stress at levels near the upper end of those experienced in the post‐capillary venule (0.1–0.6 Pa)^[^ ^26^, ^27^ ^]^ is sufficient to achieve flow conditioning.

A second practical question we addressed is the duration of EECM‐BMEC alignment following a cessation of flow. Most µSiM‐based assays require that we stop flow to process the chip in end‐point measurements. Some, such as neutrophil transmigration measurements, require that live EECM‐BMECs retain shear‐conditioned phenotypes for at least 1 h after flow.^[^ ^8^ ^]^ Having not seen the question of the durability of endothelial shear conditioning addressed elsewhere in the literature, we monitored “dealignment” of EECM‐BMECs exposed to 0.5 Pa shear for 48 h. We followed the alignment of living monolayers until the number of cells aligned with the original flow (± 30°) returned to pre‐shear baseline levels. We found that complete dealignment took 2 days, with changes detectable as soon as 3 h post‐flow. No changes in cell alignment were detectable within the first 2 h after stopping flow. With these results as a guide, we conducted all endpoint assays on flow‐conditioned chips within a 2 h post‐flow window.

### 2.3 Shear Conditioning Promotes Barrier Resilience to Low but Not High Concentrations of Circulating Cytokines

**Figure 3:**
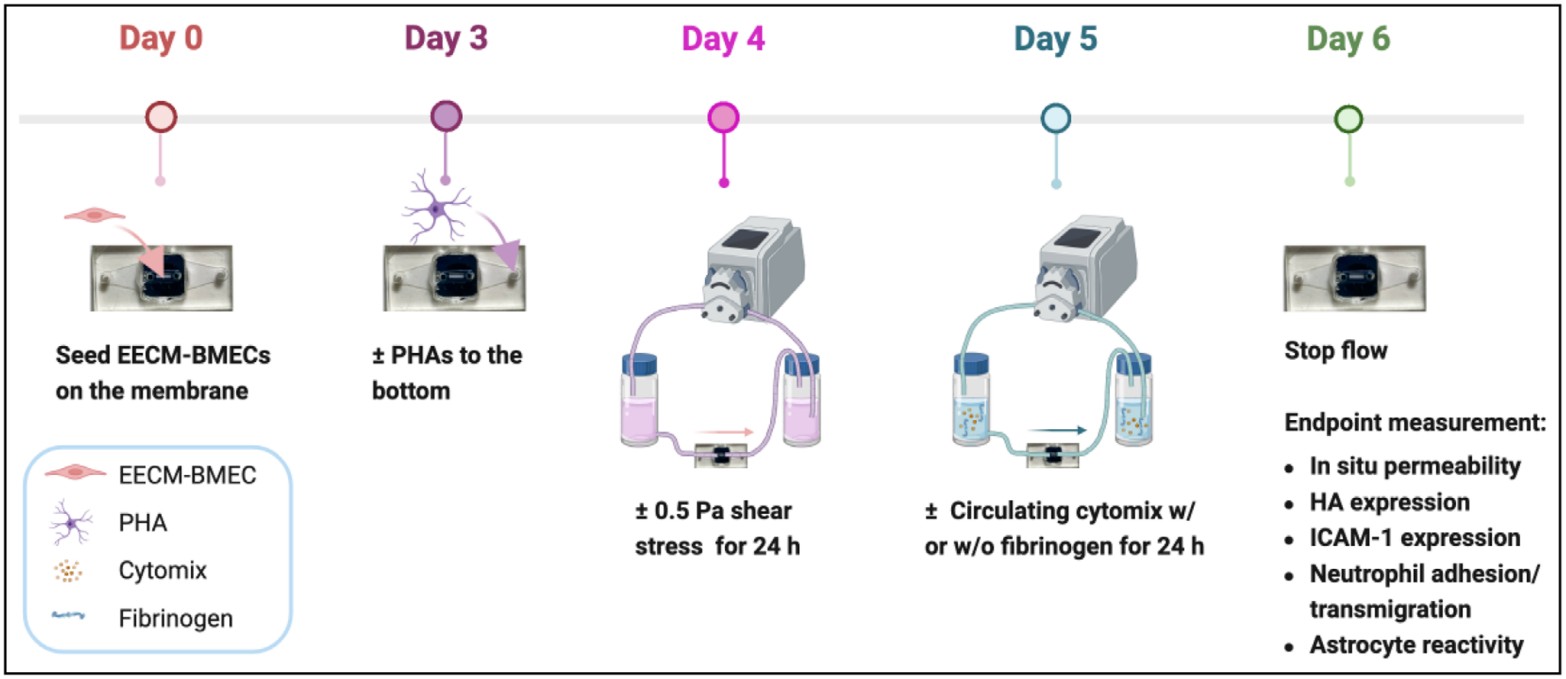
Experimental Timeline for Inflammatory Challenge in the Fluidic µSiM‐BBB Model. On Day 0, EECM‐BMECs (40 000 cells cm^−^^2^, iPSC‐derived brain microvascular endothelial cells) are seeded into collagen IV/fibronectin‐coated (400/100 µg mL^−1^) luminal channels. **On Day 3**, PHAs (33 000 cells cm^−^^2^) are seeded into collagen I/fibronectin‐coated (100/50 µg mL^−1^) abluminal channels to establish cocultures, while monoculture controls retain EECM‐BMECs only. **On Day 4**, laminar flow (0.5 Pa) is introduced in the fluidic groups to the confluent EECM‐BMEC barrier, while static groups remain in no‐flow conditions. **On Day 5**: 1) Inflammatory fluidic groups are simulated by replacing the medium in the flow circuit with 5 mL of either 10 or 50 pg mL^−1^ cytomix for cytomix stimulation groups, or 50 pg mL^−1^ cytomix + 2.5 mg mL^−1^ fibrinogen for cytomix + fibrinogen combined stimulation groups. Flow was maintained for an additional 24 h. 2) For flow control groups, a stable 0.5 Pa shear stress is sustained until Day 6 without inflammatory stimulation. 3) For static inflammatory groups, EECM‐BMEC medium is replaced with medium either supplemented with 10 or 50 pg mL^−1^ cytomix or 50 pg mL^−1^ cytomix + 2.5 mg mL^−1^ fibrinogen in both luminal flow channels and atop the inserts, while PHAs remained in astrocyte growth medium (AGM). 4) For static control groups, EECM‐BMECs (and PHAs if in coculture) are maintained in their own culture media and replenished daily until Day 6. **On Day 6**, flow is stopped for further measurements, including in situ permeability, hyaluronic acid (HA) expression, ICAM‐1 expression, neutrophil trafficking, and astrocyte reactivity.

To investigate the barrier protective potential of hemodynamic conditioning under inflammatory conditions, confluent EECM‐BMEC monolayers were exposed to 0.5 Pa shear stress for 48 h following 4 days of static culture. After 24 h of flow, acute systemic inflammation was simulated in a subset of devices by adding a cytokine cocktail (10 or 50 pg mL^−1^ cytomix, TNF‐α/ IL‐1β/ IFN‐γ) to the reservoir of the flow circuit (Figure 1C). Barrier function in these devices were compared to non‐inflammatory flow controls or static controls using the in situ version of a small molecule permeability assay using Lucifer Yellow (MW = 457 Da) as the fluorescent tracer as described,^[^ ^8^ ^]^ but with slight modifications to account for the use of a flow insert (see Experimental Section; Figure S2, Supporting Information). Static control groups were cultured for 6 days or exposed to cytokines via media supplementation 24 h prior to permeability assessment (**Figure** **3**).

**Figure 4:**
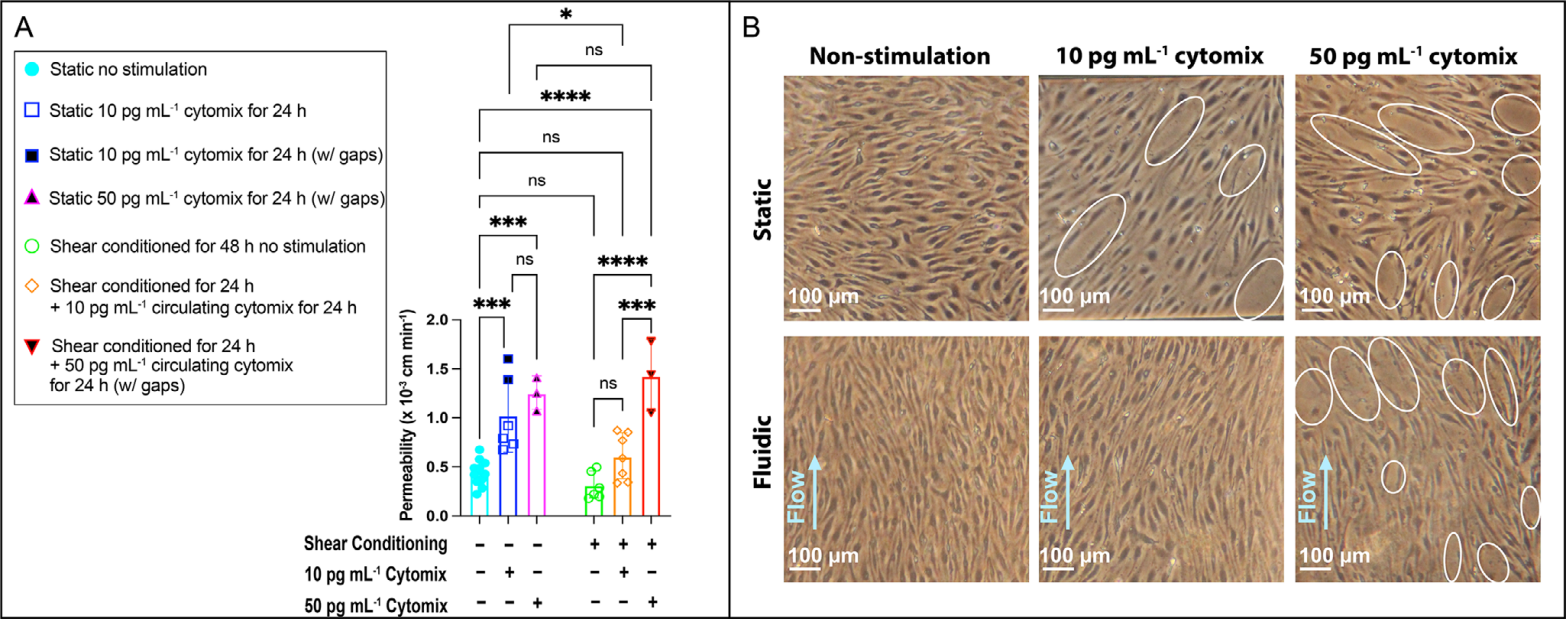
Shear conditioning enhances of EECM‐BMEC monoculture barrier resilience to inflammatory challenges. A) Quantification of barrier permeability in EECM‐BMEC monocultures under static and shear conditions (0.5 Pa) with low‐dose (10 pg mL^−1^) or high‐dose (50 pg mL^−1^) cytomix treatment. Shear stress significantly preserved barrier integrity at low doses of cytomix but failed to protect against high doses, as indicated by the increased permeability in high‐dose shear‐treated groups compared to the non‐stimulated controls. Data points filled with black indicate permeability values from barriers with visible gaps, reflecting significant structural damage. Statistical significance was assessed using a one‐way ANOVA with Tukey's post‐hoc: ****p < 0.0001, ***p < 0.001, **p < 0.01, *p < 0.05, ns > 0.05. Data are presented as mean ± standard deviation (SD), with n = 3–12 per condition. B) Representative phase‐contrast images of EECM‐BMEC monolayers cultured under static or fluidic (shear‐conditioned) conditions, with or without inflammatory cytokine stimulation (cytomix, 10 or 50 pg mL^−1^ for 24 h). White circles indicate barrier gaps observed following cytomix treatment. Gaps are defined as macroscopically visible discontinuities (≈20 µm and larger) in the endothelial monolayer when viewed in live phase contrast microscopy (10× objectives). They are characterized by loss of cell–cell contact and absence of nuclei within the void area and have a concurrent loss of junction stain (Figure S3, Supporting Information). Scale bar = 100 µm.

Results demonstrated that both unstimulated shear‐conditioned and static barriers exhibited low baseline permeability Pe*_LY_* < 0.6 × 10^−3^ cm min^−1^, **Figure** **4**A), consistent with the “tight barrier” we previously reported for EECM‐BMECs.^[^ ^8^, ^36^ ^]^ The reduction in permeability with shear‐conditioning is notable. Although the difference does not achieve statistical significance at *p < 0.05, it is significant at *p* < 0.1. The shear‐conditioned barriers were also more reproducible in their barrier function, as indicated by lower standard deviations (as a percentage of the mean). We interpret these results as evidence that shear conditioning improves baseline EECM‐BMECs barrier function in the µSiM beyond the already “tight” phenotype seen in the absence of flow.

Importantly, in response to a low‐dose cytokine challenge (10 pg mL^−1^ cytomix, mimicking early sepsis^[^ ^41^, ^42^ ^]^), shear‐conditioned barriers remained “tight” while static barriers became leaky (∗∗∗*p* < 0.001, Figure 4A). Phase contrast imaging also confirmed monolayer continuity under this condition (Figure 4B), suggesting shear priming stabilizes EECM‐BMEC barrier against mild inflammatory insult. In contrast, an inflammatory challenge consistent with severe cytokine storms (50 pg mL^−1^^[^ ^43^, ^44^ ^]^) overwhelmed both static and shear‐enhanced EECM‐BMEC monolayers, resulting in disrupted barriers (Figure 4B). Pe*_LY_* in shear‐conditioned monolayers surged and was statistically indistinguishable from static inflammatory groups (*p* > 0.05). Our data suggest a cytokine concentration threshold exists somewhere between 10 and 50 pg mL^−1^ where shear conditioning becomes ineffective in bolstering the capacity of EECM‐BMEC barriers to withstand inflammatory challenges in the µSiM.

### 2.4 Shear Stress Stabilizes Hyaluronic Acid (HA) and Confers Barrier Resilience Against Low‐Dose Inflammation

**Figure 5:**
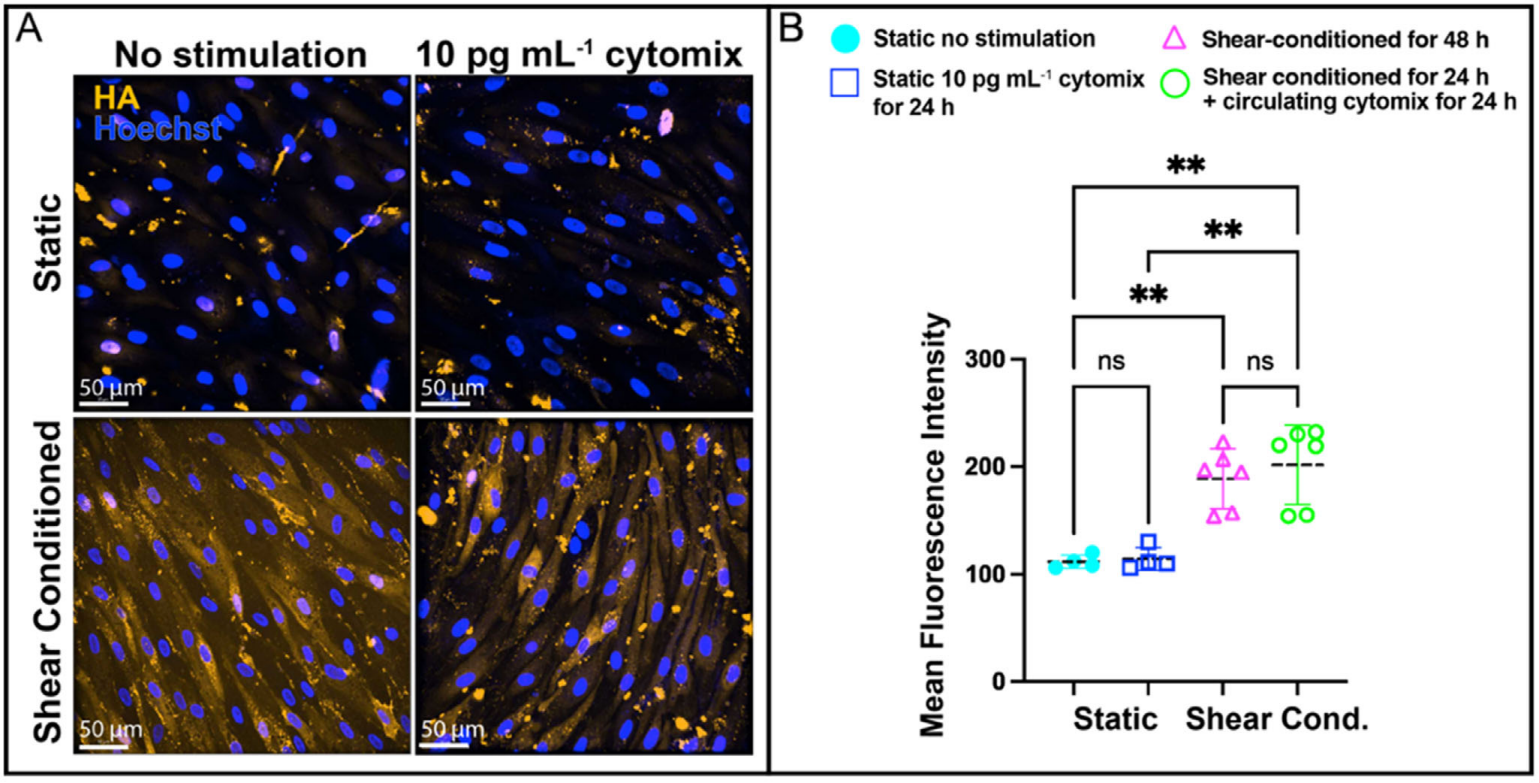
Shear Conditioning Enhances Glycocalyx Expression in EECM‐BMECs. A) Representative immunofluorescence images of HA (yellow) expression in EECM‐BMECs cultured in the µSiM model under static versus fluidic (shear‐conditioned) conditions, with and without low‐dose inflammatory cytokine stimulation (10 pg mL^−1^ cytomix for 24 h). Hoechst nuclear stain (blue) marks cell nuclei. Under static conditions, HA expression remained at baseline, with no change under inflammatory stimulation. In contrast, fluidic control conditions led to an obvious increase in HA expression compared to static controls. HA levels in fluidic inflammatory conditions measured as overall mean fluorescence intensities across the image were comparable to fluidic controls. Fluorescence images were acquired using an Andor Spinning Disc Confocal Microscope. Scale bar = 50 µm. B) Quantification of HA mean fluorescence intensity (MFI) across the image, representing glycocalyx HA expression levels across different experimental conditions: static non‐stimulated control (cyan), static inflammatory (barrier without gaps, blue), shear‐conditioned for 48 h (magenta), and pre‐shear‐conditioned for 24 h followed by circulating cytokine exposure for 24 h (green). Fluidic controls exhibited significantly higher HA expression compared to static controls, while fluidic inflammatory conditions maintained similar HA levels as fluidic controls, indicating a shear‐induced stabilization effect. Statistical significance was assessed using one‐way ANOVA with Tukey's post‐hoc: ∗∗∗∗*p* < 0.0001, ∗∗∗*p* < 0.001, ∗∗*p* < 0.01, ∗*p* < 0.05, ns > 0.05. Data were pooled from all cells under each condition for statistical analysis (n = 3–4 per condition) and are presented as mean ± SD.

The endothelial glycocalyx, a mechanosensitive barrier critical for vascular integrity,^[^ ^45^, ^46^, ^47^ ^]^ is known to exhibit an increase in response to shear stress.^[^ ^48^, ^49^ ^]^ To dissect how shear conditioning modulates the endothelial glycocalyx during inflammation, we quantified overall HA immunofluorescence (**Figure** **5**). HA is a glycosaminoglycan component of the glycocalyx with a role in mechanotransduction.^[^ ^45^, ^46^, ^47^ ^]^ We quantified overall HA expression under static conditions, physiological shear stress (0.5 Pa) and low‐dose treatment with cytomix (10 pg mL^−1^). Static, non‐stimulated monolayers exhibited baseline HA levels, consistent with low glycocalyx in the absence of hemodynamic forces.^[^ ^48^, ^49^, ^50^ ^]^ Exposure to low‐dose cytomix under static conditions did not alter HA levels significantly (*p* > 0.05). In contrast, shear conditioning alone elevated HA by 69% (∗∗*p* < 0.01).^[^ ^48^, ^49^, ^50^ ^]^ Under fluidic inflammatory conditions (shear conditioning + cytomix), HA levels remained comparable to shear conditioning alone (*p* > 0.05) and significantly exceeded static controls (∗∗*p* < 0.01).

### 2.5 Shear Stress Attenuates Cytokine‐Induced Endothelial Activation Evidenced by Reduced ICAM‐1 Expression

**Figure 6:**
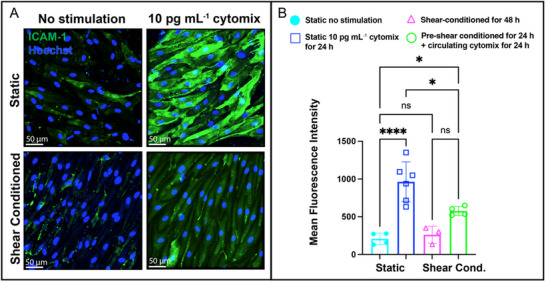
Shear conditioning attenuates endothelial ICAM‐1 expression in EECM‐BMECs induced by low‐dose cytokines. A) Representative immunofluorescence images of ICAM‐1 expression (green) in EECM‐BMECs cultured in the µSiM model under static versus fluidic (shear‐conditioned) conditions, with and without low‐dose inflammatory cytokine stimulation (10 pg mL^−1^ cytomix for 24 h). Hoechst nuclear stain (blue) marks cell nuclei. Under static conditions, inflammatory stimulation markedly increased ICAM‐1 expression, whereas shear conditioning attenuated this response, preserving a lower baseline level of ICAM‐1 expression. Fluorescence images were acquired using an Andor Spinning Disc Confocal Microscope. Scale bar = 50 µm. B) Quantification of ICAM‐1 maximum fluorescence intensity via Z‐stack projection under different experimental conditions: static non‐stimulated control (cyan), static inflammatory (blue), shear‐conditioned for 48 h (magenta), and pre‐shear‐conditioned for 24 h followed by circulating cytokine exposure for 24 h (green). Cytokine stimulation significantly increased ICAM‐1 expression under static conditions (∗∗∗∗*p* < 0.0001), whereas shear conditioning for 48 h dampened this inflammatory upregulation, maintaining significantly lower ICAM‐1 expression compared to the static inflammatory group (*p* < 0.05). Pre‐shear conditioning followed by cytokine exposure resulted in intermediate ICAM‐1 expression, trending lower than the static inflammatory group but not significantly different from the shear‐conditioned control. These findings indicate that physiological shear stress protects against cytokine‐induced endothelial activation. Statistical significance was assessed using one‐way ANOVA with Tukey's post‐hoc: ∗∗∗∗*p* < 0.0001, ∗∗∗*p* < 0.001, ∗∗*p* < 0.01, ∗*p* < 0.05, ns > 0.05. Data are presented as mean ± SD (n = 3–6 per condition).

We next investigated if the anti‐inflammatory effects of shear stress extended to endothelial activation. To assess inflammatory endothelial responses, we quantified the upregulation of intercellular adhesion molecule‐1 (ICAM‐1)—a leukocyte adhesion marker that is reliably upregulated in EECM‐BMEC cells stimulated with cytomix.^[^ ^7^, ^9^, ^36^ ^]^ ICAM‐1 immunostaining and quantification (**Figure** **6**A,B) revealed that static monolayers exposed to low‐dose cytomix (10 pg mL^−1^) exhibited a 4.63‐fold increase in ICAM‐1 expression compared to unstimulated controls (∗∗∗∗*p* < 0.0001, Figure 6B). Shear‐conditioned unstimulated monolayers maintained near‐basal ICAM‐1 levels. Shear‐conditioned monolayers exposed to low‐dose cytomix (10 pg mL^−1^; 24 h conditioning followed by static cytokine challenge) yielded intermediate ICAM‐1 expression—a 40% reduction compared to static cytomix groups (∗*p* < 0.05; Figure 6B).

### 2.6 Shear Conditioning Dampens Neutrophil Transmigration Response Following an Inflammatory Challenge

**Figure 7:**
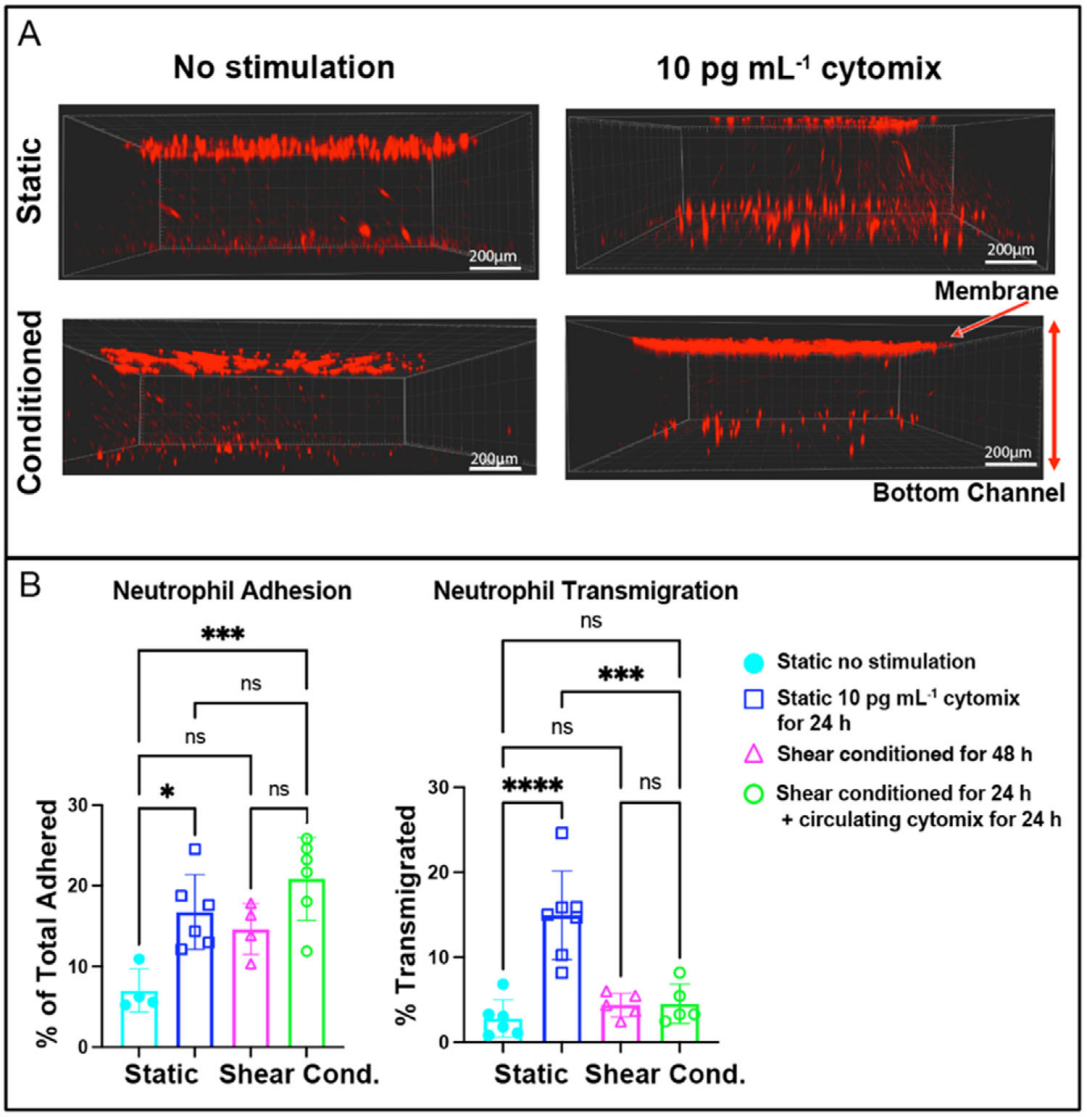
Shear conditioning dampens low‐dose cytokine‐induced neutrophil transmigration but not adhesion. A) Representative confocal *z*‐projection images of neutrophil adhesion and transmigration across the EECM‐BMEC barrier under static and fluidic (shear‐conditioned) conditions. Non‐stimulated (baseline) and low‐dose inflammatory cytokine (10 pg mL^−1^ cytomix) conditions were tested. Neutrophils are shown in red, highlighting their adhesion to the endothelial monolayer (on the nanomembrane) and migration through the membrane into the bottom channel. Fluorescence images were acquired using an Andor Spinning Disc Confocal Microscope. Scale bar = 200 µm. B) Quantification of adhered (*left*) and transmigrated (*right*) neutrophils under different experimental conditions: static non‐stimulated control (cyan), static inflammatory (blue), shear‐conditioned for 48 h (magenta), and pre‐shear‐conditioned for 24 h followed by circulating cytokine exposure for 24 h (green). Neutrophil adhesion was significantly increased in both static inflammatory conditions and shear‐conditioned groups, while neutrophil transmigration was markedly suppressed by prior shear exposure despite inflammatory stimulation. Statistical significance was assessed using one‐way ANOVA with Tukey's post‐hoc: ∗∗∗∗*p* < 0.0001, ∗∗∗*p* < 0.001, ∗∗*p* < 0.01, ∗*p* < 0.05, ns > 0.05. Data are presented as mean ± SD (n = 4–7 per condition).

To evaluate functional consequences of shear‐mediated glycocalyx enhancement and the muted upregulation of ICAM‐1, we quantified neutrophil adhesion and transmigration under low‐dose cytomix (10 pg mL^−1^) on static versus shear‐conditioned EECM‐BMEC monolayer. Static EECM‐BMEC monolayers exposed to cytomix exhibited a 2.4‐fold increase in neutrophil adhesion compared to unstimulated controls (∗*p* < 0.05, **Figure** **7**B), aligning with cytokine‐driven ICAM‐1 upregulation (Figure 6B). Shear‐conditioned EECM‐BMECs (48 h, 0.5 Pa) also displayed elevated baseline adhesion (*p* > 0.05), though not statistically significant, and further increased neutrophil adhesion under cytomix exposure (∗∗∗*p* < 0.001, Figure 7B). This elevated neutrophil adhesion post shear, despite suppressed ICAM‐1, suggests alternative adhesive mechanisms are at play, such as shear‐sensitive P‐selectin glycoprotein ligand‐1 (PSGL‐1),^[^ ^51^, ^52^ ^]^ platelet endothelial cell adhesion molecule‐1 (PECAM‐1),^[^ ^53^ ^]^ single‐chain type‐1 glycoprotein (CD99),^[^ ^54^ ^]^ and junctional adhesion molecule (JAM).^[^ ^55^ ^]^

In contrast to neutrophil adhesion, transmigration was suppressed by shear conditioning. While static cytomix‐challenged EECM‐BMEC monolayers permitted a 3.2‐fold increase in neutrophil diapedesis, shear‐conditioned barriers retained baseline transmigration levels (Figure 7B). When shear‐conditioned EECM‐BMEC barriers were subjected to cytomix exposure, neutrophil transmigration remained suppressed (vs static inflammatory; ∗∗∗*p* < 0.001), correlating with preserved barrier integrity (*P* = 0.59 ± 0.23 × 10^−3^ cm min^−1^; Figure 4A). It is also important to note that neutrophils were introduced to the system after flow cessation to isolate the effects of shear‐conditioned EECM‐BMECs in regulating adhesion and transmigration, independent of direct shear forces on neutrophils.

In a complementary study we assayed CXCL8 secretion into the basal compartment of the µSiM under static and flow conditions, with and without luminal cytokine stimulation (Figure S4, Supporting Information). CXCL8 was selected because it is a potent neutrophil chemokine secreted by endothelial cells during inflammation. We focused on the basal compartment because our previous work demonstrated that basal chemokine levels are the primary driver of neutrophil transmigration across model barriers.^[^ ^56^, ^57^ ^]^ Moreover, unlike the apical compartment, the basal compartment of the fluidic µSiM is unaffected by flow and is not diluted by recirculating media, enabling direct comparison of secretion levels between static and flow experiments. Our results showed substantial basal CXCL8 secretion after inflammatory challenge under static conditions, which was completely abolished under physiological flow. This pattern closely mirrors the neutrophil transmigration profile on pre‐conditioned endothelium, both with and without luminal cytokine challenge. Consistent with our earlier work in HUVECs,^[^ ^56^, ^57^ ^]^ these findings confirm that basal CXCL8 levels are a key driver of neutrophil transmigration. The observation that both basal CXCL8 secretion and neutrophil transmigration are diminished following BMEC shear conditioning provides additional mechanistic evidence for the anti‐inflammatory effects of shear conditioning.

### 2.7 PHAs Exhibit Dose‐Dependent Reactive Gliosis to Cytomix Stimulation as Neuroinflammation “Sensor” in the µSiM‐BBB

Having established the critical role of shear stress in supporting endothelial barrier function under inflammatory conditions, we next asked if we could simulate a circulating inflammatory challenge strong enough to “break the barrier” and trigger a neuroinflammatory response on the “brain side” of the µSiM‐BBB. To model this, we added primary human astrocytes (PHAs) to the bottom component of the µSiM‐BBB as dynamic “sensors” of neuroinflammation. This allowed us to leverage the clear morphological changes in astrocytes during reactive gliosis as our marker of “brain side” inflammation triggered by injurious factors circulating on the “blood side” of the chip. Reactive gliosis is a hallmark of astrocytic activation in neurodegenerative and neuroinflammatory disorders.^[^ ^30^, ^31^, ^32^ ^]^ Astrocytes, positioned at the neurovascular interface, respond to BBB‐derived inflammatory mediators (e.g., cytokines, fibrinogen) in a manner that exacerbates barrier breakdown and can lead to neuronal damage.^[^ ^33^, ^34^, ^58^ ^]^

**Figure 8:**
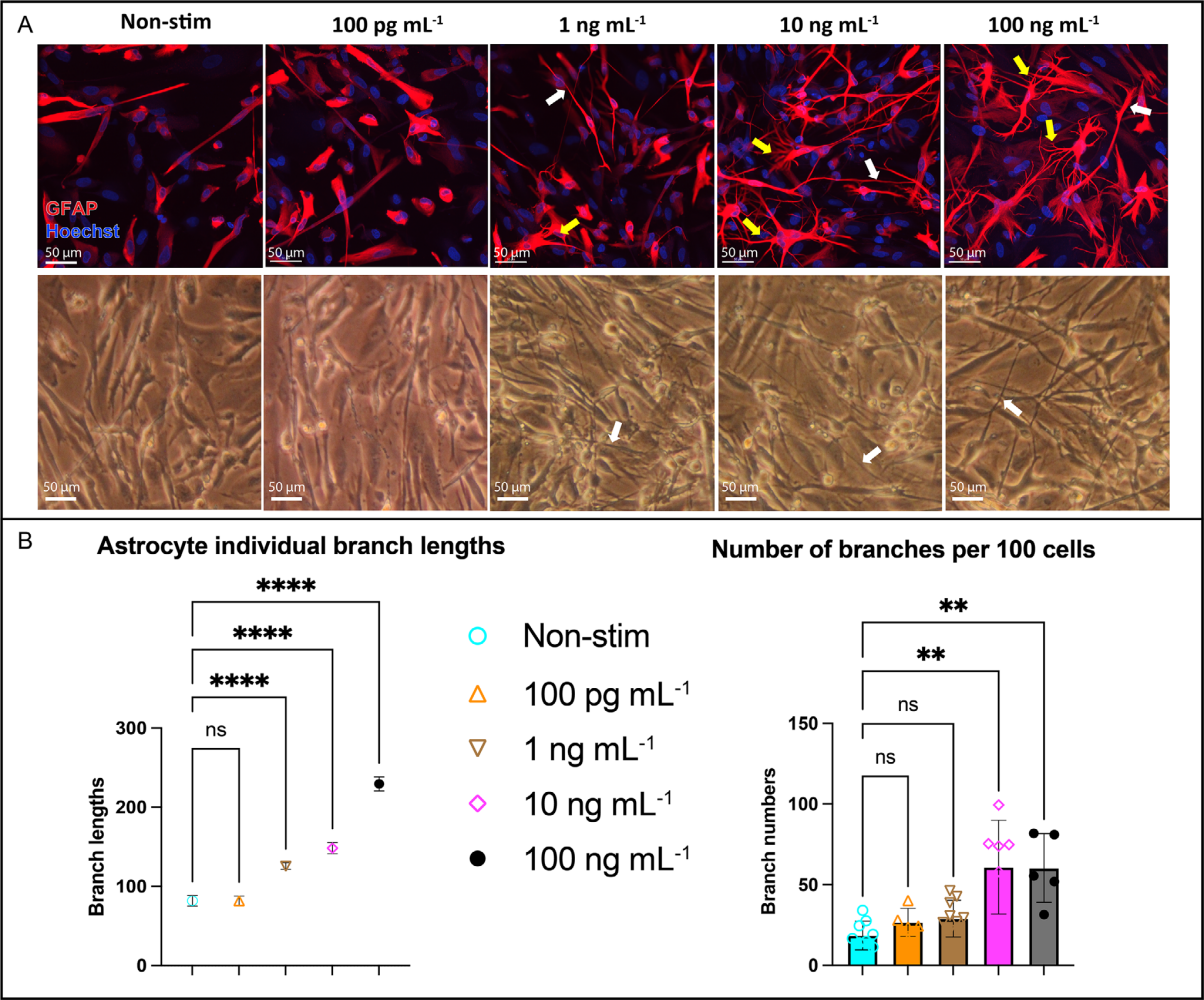
Dose‐dependent activation of PHA monoculture in response to increasing cytomix concentrations highlights their potential as inflammatory “sensors.” A) GFAP immunostaining (red) and Hoechst nuclear counterstaining (blue) of PHAs after 24‐h exposure to escalating doses of cytomix (0.1, 1, 10, and 100 ng mL^−1^). Cytomix treatment induces a dose‐dependent morphological transformation, with hallmark features of reactive astrogliosis, including hypertrophic cell bodies, long branches (white arrows), and higher branching complexity (yellow arrows). Scale bar = 50 µm. (A, second row) Phase‐contrast microscopy images of PHAs post‐cytomix treatment, showing similar dose‐dependent morphological changes, consistent with astrocyte activation. White arrows indicate elongated‐branch astrocytes: individual astrocytes with GFAP⁺ processes extending > 100 µm from the soma (vs control: 81.80 ± 0.29 pixels). Yellow arrows highlight increased branching complexity: ≥ 3 secondary branches per primary process. Scale bar = 100 µm. B) Quantification of astrocyte branching complexity via skeleton analysis of GFAP^+^ PHAs, demonstrating a significant increase in branch length at cytomix concentrations ≥ 1 ng mL^−1^ and a significant increase in the number of branches (per 100 cells, total cell numbers per device shown in Table S1, Supporting Information) at concentrations ≥ 10 ng mL^−1^. Statistical significance was assessed using one‐way ANOVA with Tukey's post‐hoc, ****p < 0.0001, ***p < 0.001, **p < 0.01, *p < 0.05, ns > 0.05. Data were pooled from all cells under each condition, then grouped for statistical analysis. Branch lengths are presented as mean ± SEM, and the number of branches are presented as mean ± SD (n = 5–8 per condition).

Before conducting experiments on PHAs in the µSiM‐BBB coculture, we titrated cytomix (0.1–100 ng mL^−1^) in PHA monoculture within the µSiM to assess their responsiveness and identify doses that elicit clear morphological changes. Immunofluorescence imaging of GFAP, a canonical marker of astrocyte activation,^[^ ^33^, ^59^ ^]^ revealed a dose‐dependent morphological transformation in PHAs (**Figure** **8**). At subthreshold cytokine levels (0.1 ng mL^−1^), quiescent morphology in both branch length and branch count compared to control (*p* > 0.05; Figure 8B). However, ≥ 1 ng mL^−1^ cytomix triggered progressive reactive gliosis, characterized by elongated processes (∗∗∗∗*p* < 0.0001; Figure 8A) but normal branched networks (*p* > 0.05 vs control; Figure 8B). Phase‐contrast imaging corroborated these findings, showing dose‐dependent increases in cellular protrusions and network density (Figure 8A), consistent with cytoskeletal remodeling reported in neuroinflammatory pathologies.^[^ ^33^ ^]^ At higher doses (10 ng mL^−1^), PHAs exhibited further morphological changes with increases in both branch length (∗∗∗∗*p* < 0.0001) and branch counts (∗∗*p* < 0.01; Figure 8B). This hyperbranched phenotype reflects the transition from physiological astrocyte activation to pathological gliosis, as observed in neuroinflammatory conditions such as multiple sclerosis and traumatic brain injury.^[^ ^59^ ^]^ Finally, at supraphysiological concentrations (100 ng mL^−1^), PHAs adopted a profoundly reactive phenotype, with GFAP^+^ networks spanning longer distances (∗∗∗∗*p* < 0.0001 vs control; Figure 8B) and dense branch counts (∗∗*p* < 0.01 vs control; Figure 8B).

While the graded response of PHAs to cytomix exposure establishes their utility as dynamic indicators of neuroinflammation in the µSiM‐BBB, the threshold for visible activation of PHAs is surprisingly high (cytomix ≥ 1 ng mL^−1^). This is much higher than plasma levels seen during sepsis and 20 folds higher than the levels that we used to disrupt shear‐conditioned EECM‐BMEC barriers in the µSiM‐BBB (50 pg mL^−1^). Thus, we would not expect the passage of 50 pg mL^−1^ cytomix across a leaky BBB to result in morphologically visible PHA activation. For this reason, we examined the combination of using circulating inflammatory cytokines—to open the BBB—with fibrinogen, a soluble plasma protein known to cause direct astrocyte activation in the brain following traumatic brain injury and sepsis.^[^ ^34^, ^60^ ^]^ Our goal was a capstone experiment to illustrate the potential of the fluidic µSiM‐BBB to simulate the inflammatory cascade connecting systemic inflammation to brain injury in a disease context.

### 2.8 Combinatorial Circulating Factors Drive Astrogliosis Downstream of BBB Disruption

**Figure 9:**
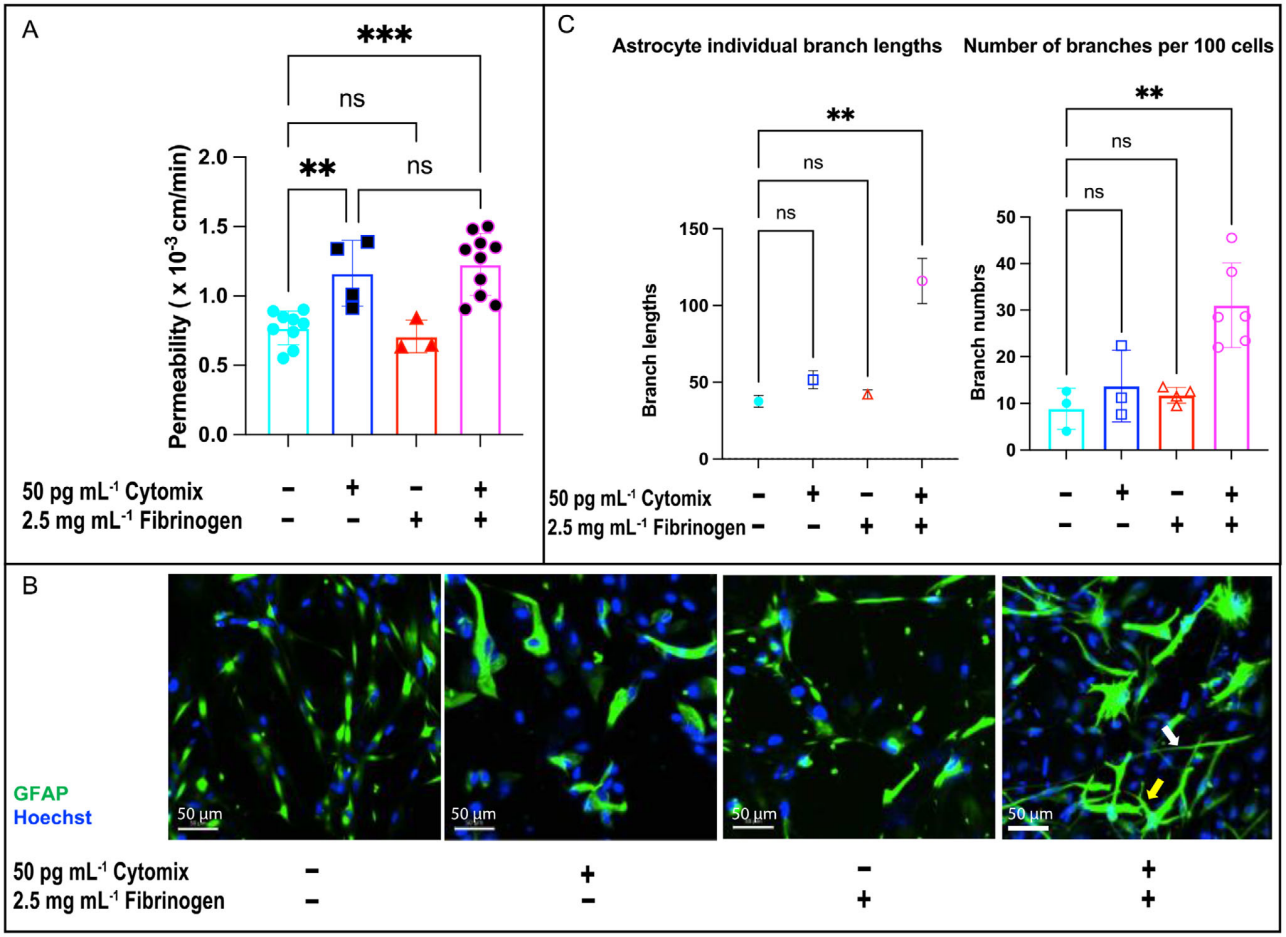
Brain side astrocyte activation requires a multi‐hit inflammatory challenge in the fluidic µSiM‐BBB. A) Barrier permeability disruption in the fluidic µSiM‐hBBB with EECM‐BMEC/PHA Co‐Cultures: Permeability was similarly increased under both 50 pg mL^−1^ cytomix alone and 50 pg mL^−1^ cytomix + 2.5 mg mL^−1^ fibrinogen conditions, indicating barrier disruption after a high dose of cytomix despite shear conditioning. Data points filled with black represent conditions where endothelial gaps were observed (Figure S5, Supporting Information), highlighting structural damage to the barrier. Statistical significance was assessed using a one‐way ANOVA with Tukey's post‐hoc: ****p < 0.0001, ***p < 0.001, **p < 0.01, *p < 0.05, ns > 0.05. Data are presented as mean ± standard deviation, with n = 3–7 per condition. B) Astrocyte Activation in Response to Combined Cytomix and Fibrinogen Treatment: While neither 50 pg mL^−1^ cytomix alone nor 2.5 mg mL^−1^ fibrinogen alone induced morphological changes in astrocytes, their combination resulted in profound astrocyte activation, as evidenced by GFAP immunostaining. Reactive astrocytes exhibited hypertrophic cell bodies, increased dendritic complexity (yellow arrow), and extensive GFAP^+^ interwoven networks (white arrow), indicative of robust astrogliosis. This activation pattern was observed under combined cytomix + fibrinogen exposure in the µSiM‐BBB coculture but was absent in non‐stimulated controls and single‐treatment groups, reinforcing the hypothesis that astrocyte activation requires a multi‐hit inflammatory insult. Scale bar = 50 µm C) Quantification of Astrocyte Activation: Skeleton analysis revealed a significant increase in astrocyte branch length and branch number per 100 cells in the combined cytomix + fibrinogen group under both static and fluidic conditions, whereas single‐treatment groups showed no significant activation compared to controls. Statistical significance was assessed using one‐way ANOVA: Statistical significance was assessed using a one‐way ANOVA: ****p < 0.0001, ***p < 0.001, **p < 0.01, *p < 0.05, ns > 0.05. Branch lengths are presented as mean ± SEM, and the number of branches is presented as mean ± SD (n = 3–6 per condition).

To model neuroinflammatory cascades triggered by severe systemic inflammation, we cultured PHAs on the floor of the bottom channel of the fluidic µSiM‐BBB (Figure 1A) and introduced both 50 pg mL^−1^ cytomix and 2.5 mg mL^−1^ fibrinogen into the circulation following 24 h of shear conditioning. Morphologically, astrocytes displayed minimal levels of activation under baseline conditions (**Figure** **9**B,C). Shear‐conditioned cocultures exposed to circulating fibrinogen alone exhibited similar barrier integrity as baseline (Figure 9A), while shear‐conditioned cocultures exposed to circulating cytomix alone exhibited significant permeability increases (∗∗*p* < 0.01, Figure 9A), without morphological changes in astrocytes (*p* > 0.05 vs control). The combined cytomix + fibrinogen exposure triggered measurable morphological changes in astrocytes (∗∗*p* < 0.01 vs fluidic control; Figure 9B,C), despite no additional barrier compromise versus cytomix alone (*p* > 0.05). Immunofluorescence confirmed endothelial junctional disruption (Figure S5, Supporting Information), enabling permeation of large fibrinogen molecules (340 kD) through the BBB to reach astrocytes, which adopted the proinflammatory phenotype. This successfully demonstrates the use of the fluidic µSiM‐BBB to simulate an acute episode of systemic inflammation leading to a marker of brain injury—the reactive astrocyte. Many more questions can now be asked of this system, including identifying threshold levels for triggering the neuroinflammatory cascade and the potential of therapeutic interventions to raise this threshold. The scope of the current study, however, is completed with the establishment of engineering elements and workflows that will enable this future work.

## 3 Discussion

The successful development of the fluidic µSiM‐BBB is a key advancement in our effort to create an in vitro tool to model the injury cascade that starts with peripheral inflammation and ends with brain damage. This sequence is seen in cases of severe sepsis,^[^ ^61^ ^]^ traumatic brain injury,^[^ ^62^ ^]^ major surgery,^[^ ^63^ ^]^ acute lung injury,^[^ ^64^ ^]^ and even as a side effect of therapies that enlist the immune system to fight cancer.^[^ ^65^, ^66^ ^]^ While the cellular and molecular details change with disease context, the transduction of peripheral inflammation to neuroinflammation inevitably involves a circulating challenge that overwhelms the protective functions of the BBB to reach and activate parenchymal cells and injure neurons.

Using microfluidics to model the introduction of inflammatory insults via a circulation is important for multiple reasons. First, the forces of fluid flow, namely shear stresses at the luminal surface, differentiate endothelial cells toward phenotypes with numerous anti‐inflammatory characteristics.^[^ ^13^, ^14^, ^15^, ^16^, ^17^ ^]^ Since vascular endothelial cells are always experiencing these forces in vivo, experiments and models that use static vascular endothelial cultures are making a choice of convenience with some known, and many unknown, compromises. Critical among the known benefits of shear conditioning of the endothelial phenotype is an enhanced barrier function, which comes about through morphological rearrangements of cells in the flow field.^[^ ^4^, ^12^ ^]^ Including fluid flow also improves our simulation of molecular and cellular delivery to the barrier tissue. For example, immune cells interact with endothelial cell surfaces by first rolling along the glycoprotein‐rich surfaces of endothelial cells (glycocalyx) before arresting and transmigrating,^[^ ^67^ ^]^ phenomena that have been replicated in the µSiM in non‐BBB contexts.^[^ ^13^, ^25^ ^]^ The enhanced mass transport from fluid flow increases the accuracy of molecular concentrations at the blood–brain interface on the chip, and enables the clearance of pro‐inflammatory responses via the circulation, as would naturally occur in vivo.

Our work here confirms shear‐induced morphological responses and barrier enhancement in BMEC‐like cells derived from iPSCs using the extended endothelial culture method (EECM).^[^ ^7^, ^39^ ^]^ This protocol, which directs differentiation through a mesodermal lineage rather than an ectodermal/epithelial one^[^ ^68^, ^69^ ^]^ results in BMEC‐like cells with appropriate vascular endothelial gene expression, including leukocyte adhesion molecules. Here, we show that shear‐conditioned EECM‐BMECs display an enhanced glycocalyx (HA) and lower permeability at baseline compared to statically grown cells. We also show that shear conditioning results in a muted expression of ICAM‐1 and neutrophil transmigration in response to the low doses of the “cytomix” inflammatory cocktail (10 pg mL^−1^ TNF‐α, IL‐1β, IFN‐γ).

Significant among our findings is that the shear‐conditioned EECM‐BMEC barrier does not transition to leaky phenotypes at a 10 pg mL^−1^ dose of cytomix as static cultures do. Unsurprisingly, very high doses of cytomix (50 pg mL^−1^) severely weaken even shear‐conditioned barriers by disrupting EECM‐BMEC continuity. Somewhere between these two doses is a new threshold for the transition from tight to leaky barrier function in the fluidic µSiM‐BBB. While we do not identify this elevated threshold here, its existence is further evidence that the shear‐conditioned EECM‐BMEC is more resilient to an inflammatory challenge. These findings affirm our efforts to develop the fluidic µSiM‐BBB and indicate that shear conditioning is a critical step for our ongoing efforts to model systemic inflammation leading to brain injury on chip.

Astrocytes are one of the early responders in the brain parenchyma in response to peripheral factors following BBB opening. At the capillary level of the brain microvasculature, astrocytes are intimately associated with microvessels formed by endothelial cells and pericytes in the neurovascular unit.^[^ ^70^ ^]^ Astrocytic end feet and their associated glia limitans matrix wrap around microvessels to reinforce the vascular wall and create the most resilient blood–tissue interface in the body.^[^ ^71^ ^]^ However, the inflammatory nexus of the brain microvasculature is the post‐capillary venule, not the capillary.^[^ ^29^, ^72^, ^73^ ^]^ Here, the astrocyte end feet and glia limitations are separable from the endothelial/pericyte barrier to form serial barriers between the blood and the brain with an interstitial perivascular space. In combination with naturally reduced blood flows in the larger post‐capillary venules, the specialized architecture of the post‐capillary venule supports homeostatic surveillance by immune cells that can traffic from blood to brain and back. Following a severe peripheral insult causing BBB breakdown, the perivascular space can act as a bioreactor that concentrates immune cells, pro‐inflammatory signals, and matrix‐degrading enzymes to overwhelm the astrocyte barrier so that an injurious mix enters the parenchyma.^[^ ^5^, ^74^ ^]^

We employed astrocytes as a “sensor” to demonstrate the potential of the fluidic µSiM‐BBB to simulate the transduction of systemic inflammation to neuroinflammation. Astrocytes are ideal cell‐based sensors of brain side inflammation because they undergo easy‐to‐visualize morphological transformations driven by the polymerization of the intermediate filament GFAP.^[^ ^33^ ^]^ On‐chip immunofluorescence shows that peripheral astrocyte processes labeled with GFAP thicken, lengthen, and increase in number as astrogliosis progresses. These same morphological changes occur in response to severe acute brain injury and advanced neurodegenerative disease.^[^ ^5^, ^59^ ^]^ Astrogliosis can progress to form a “scar tissue” that has been associated with cognitive impairment,^[^ ^75^ ^]^ but may provide short‐term protection by slowing immune cell infiltration.^[^ ^59^, ^76^ ^]^ Thus, the convenient morphological changes we are tracking in astrocytes in our demonstration of chip function are also disease‐relevant markers of brain injury as an outcome of systemic inflammation.

While astrocytes are sensitive to the pro‐inflammatory factors, we use to disrupt barrier function in the µSiM‐BBB, the concentrations that elicit measurable morphological changes are an order of magnitude higher than those that cause barrier disruption. Thus, simply introducing pro‐inflammatory cytokines to the model circulation and allowing them to leak from the blood side to the brain side of the chip does not cause our astrocyte “sensor” to respond. For this reason, we also added the fibrinogen to the flow circuit at concentrations found in serum (2.5 mg mL^−1^). While fibrinogen alone did not disrupt the BBB in shear‐conditioned EECM‐BMECs, in combination with barrier‐disrupting levels of cytokines (50 pg mL^−1^) it did induce reactive astrocyte phenotypes. Fibrinogen is a carrier of latent TGF‐β, which is converted to an active form on the astrocyte surface.^[^ ^34^ ^]^ While there are several possible sources of latent TGF‐β in our experiment, the activated EECM‐BMEC is an intriguing possibility, given the absence of astrocyte activation in PHA monocultures exposed to combined cytomix and fibrinogen (Figure S6, Supporting Information). A question for future work, a role for EECM‐BMEC activation in astrocyte activation would point to a complex cascade linking blood side to brain side inflammation that involves cellular crosstalk and infiltration in addition to BBB breakdown.

Future work will also advance our model by adding pericytes to the BBB to complete the core neurovascular unit and further bolster barrier resilience. A fully isogenic culture is a goal that will allow controlled studies on the influence of genetics on brain injury as an outcome of systemic inflammation, potentially in patient‐specific models. More distant goals involve adding more “brain” to the µSiM‐BBB, notably microglia and neurons. To build these advanced systems so that they emulate human disease mechanism(s) and are predictive of therapeutic interventions requires stepwise developments such as this one. Here, we establish that fluid flow and shear‐conditioned EECM‐BMECs should be an essential element of these future models.

## 4 Experimental Section

### µSiM Components and Assembly—µSiM Components

The platform integrated two nanomembrane types from SiMPore, Inc.: 1) Nanoporous silicon nitride (NPN) membranes (models NPSN100‐1L): 100 nm thickness, 50 nm pores, 15% porosity; and 2) Dual‐scale membranes (model NPSN100‐MP‐1L‐3.0LP). Dual‐scale membranes retain NPN nanoporosity with added 3 µm pores [0.625% porosity] to enable leukocyte transmigration. Both membrane types are fabricated on 5.4 × 5.4 × 0.3 mm silicon chips with a central 2 × 0.7 mm membrane window (0.1 µm thickness).^[^ ^77^ ^]^ The components (Figure 1A) and assembly process of the µSiM device utilized in this study, comprising two laser‐cut acrylic components (ALine Inc., Signal Hill, CA) and silicon nitride nanomembranes (SiMPore, Inc., West Henrietta, NY), are consistent with those detailed in the previous work.^[^ ^8^, ^78^ ^]^ Component 1 (top well) and component 2 (bottom channel) were mass‐produced via laser cutting and lamination, enabling scalable fabrication of hundreds to tens‐of‐thousands of devices per production run. Protective adhesive layers ensured sterility during shipping and storage, which were removed prior to assembly.

### µSiM Components and Assembly—Open‐Well µSiM Device Assembly

Open‐well µSiM devices were assembled in a sterile biosafety cabinet following a layer‐by‐layer protocol as described in previous work.^[^ ^8^, ^13^, ^78^ ^]^ Briefly, component 1 (top well) and component 2 (bottom channel) were bonded to the silicon chip via pressure‐activated adhesion, creating luminal and abluminal compartments separated by the nanomembrane, facilitating communication of soluble factors across a barrier. All devices were UV‐sterilized (20 min) prior to cell culture.

### Polydimethylsiloxane (PDMS) Flow Inserts

Studies used both “handmade” and “manufactured” PDMS flow inserts, which perform identically in the µSiM chip. The handmade PDMS flow inserts (Figure 1A) were fabricated using standard soft lithography techniques, as adapted from the previous publication.^[^ ^13^ ^]^ Photolithography: SU‐8 3050 photoresist (Kayaku Advanced Materials) was spin‐coated onto a 100 mm silicon wafer (University Wafers), soft‐baked (95 °C, 45 min), UV‐exposed (250 mJ cm^−^^2^ through a clover‐shaped photomask), post‐baked (95 °C, 5 min), and developed. Mold Assembly: A laser‐cut acrylic divider (3.3 mm thickness) was adhered to the wafer using pressure‐sensitive adhesive (3M MP467) to define flow channels. PDMS Casting: Degassed PDMS (Sylgard 184, 10:1 w/w base‐to‐catalyst) was poured, cured (75 °C, 12 h), demolded, and port‐punched (1 mm biopsy punch). The entire process, including device assembly, required ≈2 days.

During the course of this project, the laboratory developed the flow insert as a commercially manufactured “peel‐and‐stick” module in collaboration with ALine Inc. (Signal Hill, CA). Validation studies confirmed functionally equivalent shear stress profiles, endothelial barrier properties, and neutrophil transmigration performance between manufactured and in‐house inserts.^[^ ^13^, ^25^ ^]^ The manufactured inserts reduce device assembly efforts from days to minutes while improving reproducibility of device assembly and use. The manufactured inserts were utilized for the later work in the project, including the EECM‐BMEC/PHA coculture studies.

### Fluidic µSiM—In‐House PDMS Flow Inserts

Assembly of fluidic µSiM devices with in‐house flow inserts was performed under sterile conditions within a biosafety cabinet, following a layer‐by‐layer protocol.^[^ ^8^, ^13^ ^]^ Prior to bonding, in‐house polydimethylsiloxane (PDMS) flow inserts were sonicated in 70% ethanol (v/v) for 30 min and rinsed with sterile water (Gibco). Open‐well µSiM devices were washed twice with 70% ethanol (100 µL per wash) and dried at 75 °C for 5 min. Both components were then subjected to UV/ozone surface activation (Novascan PSD Pro Series UV/Ozone Cleaner, Novascan Inc., Boone, IA; 15 min, 254 nm UV) to enhance bonding efficacy. The PDMS flow insert was manually aligned over the silicon nitride membrane, ensuring the membrane was precisely centered with the flow channels (5.0 mm length × 1.5 mm width) without edge contact. Gentle pressure was applied to non‐membrane areas using a sterile handling tweezer (Techni‐Tool, USA) to achieve hermetic sealing. Bonded devices were annealed at 75 °C overnight to stabilize interfacial adhesion and sterilized via UV for 20 min prior to cell culture.

### Fluidic µSiM—Manufactured Peel‐and‐Stick Inserts

Commercially manufactured inserts (ALine Inc., Signal Hill, CA)^[^ ^25^ ^]^ were ethanol‐sterilized (70% spray) and UV‐treated (20 min) before assembly. Protective liners were removed, and inserts were manually aligned to the µSiM membrane same as the in‐house PDMS flow inserts. Sealing was achieved by applying uniform pressure to the nonporous pressure‐sensitive‐adhesive area. Bonded devices were UV‐sterilized (20 min) and rinsed twice with sterile distilled water before use.

### Flow Circuit Configuration

A custom‐designed flow circuit (Figure 1C) was constructed to facilitate the circulation of the medium across the µSiM‐BBB model, similar to previous work.^[^ ^13^, ^25^ ^]^ The circuit comprised commercially available components: sample collection vials (BKMAM Lab, USA), Gauge 21 NT dispensing tips (Jensen Global, USA), and 0.5 × 0.86 mm microflow tubing (Langer Instruments, USA). Flow circulation was maintained using a peristaltic pump (Langer Instruments, USA), ensuring consistent shear stress across the EC layer.

### Inflammatory Stimulation Protocols—Cytomix Preparation

Sepsis‐like cytokine storm conditions were simulated by supplementing EECM‐BMEC or PHA culture media with a pro‐inflammatory cytokine cocktail (cytomix) composed of recombinant human TNF‐α (R&D Systems, 210‐TA), IFN‐γ (R&D Systems, 285‐IF), and IL‐1β (R&D Systems, 201‐LB) at equimolar concentrations. Final cytomix doses of 10 pg mL^−1^ (low) or 50 pg mL^−1^ (high) were prepared in sterile culture media.

### Inflammatory Stimulation Protocols—Combined Cytomix + Fibrinogen Stimulation

For neuroinflammatory simulations, EECM‐BMEC media was supplemented with cytomix and fibrinogen. Lyophilized human fibrinogen (plasminogen‐depleted, Sigma–Aldrich, F3879) was reconstituted in sterile distilled water (Gibco) to a stock concentration of 25 mg mL^−1^. The stock fibrinogen solution and cytomix were then diluted into EECM‐BMEC or PHA (only for PHA monoculture stimulation) media to achieve final concentrations of 50 pg mL^−1^ for cytomix and 2.5 mg mL^−1^ for fibrinogen, reflecting physiological levels.^[^ ^43^, ^44^, ^60^, ^79^ ^]^

### Cell Culture Protocols—General Conditions

All cell cultures were maintained at 3 7 °C under 5% CO_2_/95% air with saturated humidity. For static culture in the µSiM, devices were housed in humidified Petri dishes to minimize evaporation.

### Cell Culture Protocols—EECM‐BMEC Differentiation and Culture

IMR90‐4 hiPSCs (WiCell, Madison, WI) were differentiated into extended endothelial culture method brain microvascular endothelial cell (EECM‐BMEC)‐like cells as previously described.^[^ ^7^, ^39^, ^80^, ^81^, ^82^, ^83^ ^]^ Briefly, hiPSCs were seeded at a density of 70 000—100 000 cells per well in 12‐well tissue culture plates and differentiated into endothelial progenitor cells (EPCs) over three days. CD31^+^ cells were isolated using magnetic‐activated cell sorting (MACS) and expanded in endothelial culture medium (hECSR: hESFM [Gibco] + 1× serum free B‐27 [Gibco], 20 ng mL^−1^ bFGF‐2 [R&D Systems], 50 µg mL^−1^ penicillin–streptomycin [Gibco]). Cells were utilized between passages 4–6.

### Cell Culture Protocols—EECM‐BMEC General Monoculture Setup

EECM‐BMECs (2 × 10⁶ cells mL^−1^, 40 000 cells cm^−^^2^) were seeded into human collagen IV (400 µg mL^−1^; Sigma–Aldrich)/fibronectin (100 µg mL^−1^; Gibco)‐coated microfluidic µSiM flow channels. hECSR medium (75 µL) was layered atop PDMS inserts to prevent evaporation. Media was refreshed daily until day 4, otherwise specified.

### Cell Culture Protocols—EECM‐BMEC for Shear Stress Conditioning and Morphological Retention

On day 4, EECM‐BMEC monocultures were exposed to laminar shear stresses of 0.1, 0.25, or 0.5 Pa (1, 2.5, or 5 dyne cm^−^^2^) for 24, 48, or 72 h using the customized fluidic circuit. Post‐flow morphological retention was monitored via live‐cell phase‐contrast images (Olympus, CK2) at 2, 6, 12, 24, 48, and 96 h after shear cessation.

### Cell Culture Protocols—EECM‐BMEC Monoculture Barrier Stimulation

hECSR was refreshed daily after seeding under the following experimental conditions: 1) Static Control Group: Maintained in hECSR without cytomix. Media was replenished daily until Day 6. 2) Static Inflammatory Groups: On Day 5, hECSR was replaced with cytomix‐supplemented media (10 or 50 pg mL^−1^ cytomix) in both flow channels and atop the inserts (75 µL). Cytomix exposure lasted 24 h prior to subsequent assays. 3) Fluidic Control Group: After 4 days of static culture, laminar flow (0.5 Pa shear stress) was initiated using fluidic circuit mentioned above and maintained for 48 h with hECSR circulation (5 mL reservoir). 4) Fluidic Inflammatory Groups: Following 24 h of flow stabilization (0.5 Pa), hECSR was replaced with cytomix‐supplemented media (10 or 50 pg mL^−1^) in the flow circuit (5 mL reservoir) and circulated for 24 h. All experiments were terminated on Day 6 for endpoint analyses. For neutrophil adhesion and transmigration assays, physiological neutrophil concentrations (3 × 10⁶ cells mL^−1^; within the human peripheral blood range of 2.5–7 × 10⁶ cells mL^−1^)^[^ ^84^ ^]^ were introduced into the luminal compartment of EECM‐BMEC barriers. Neutrophil interactions were quantified after a 1.5‐h incubation at 37 °C under 5% CO_2_ using confocal microscopy (Andor Spinning Disk Confocal with 10× objective).

### Cell Culture Protocols—Primary Human Astrocyte Monoculture and Stimulation

Clonetics normal human astrocytes (Lonza Biosciences, passages 2–4) were seeded at a density of 33 000 cells cm^−^^2^ onto µSiM open wells precoated with rat tail collagen I (16.6 µg cm^−^^2^; Sigma–Aldrich)/ bovine fibronectin (5 µg cm^−^^2^; Corning).^[^ ^8^, ^85^ ^]^ Coatings were incubated for 1 h at 37 °C prior to cell seeding to ensure uniform substrate adhesion. Cells were maintained in astrocyte growth medium (AGM BulletKit, Lonza, CC‐3186) under static conditions at 37 °C/5% CO_2_. To model systemic inflammation‐driven cytokine leakage into the brain parenchyma, cytomix was introduced to the “blood” compartment (bottom channel) of the µSiM. AGM BulletKit medium was supplemented with cytomix at final concentrations of 0 (negative control), 100 pg mL^−1^, 1 ng mL^−1^, 10 ng mL^−1^, and 100 ng mL^−1^. For static µSiM devices, cytomix‐supplemented AGM (20 µL) was added to the bottom channel, while fresh AGM (100 µL) was retained in the “brain” compartment (open well) to establish a blood‐to‐brain cytokine diffusion. This configuration recapitulates physiological cytokine translocation across the BBB, mimicking systemic inflammation‐driven parenchymal exposure.^[^ ^2^ ^]^ To model combined inflammatory leakage into the brain parenchyma, a 50 pg mL^−1^ cytomix + 2.5 mg mL^−1^ fibrinogen mixture was introduced to the “blood” compartment of the µSiM, mimicking coagulation‐associated inflammatory signaling observed in sepsis and trauma‐related neurovascular injury.

### Cell Culture Protocols—EECM‐BMEC/PHA Coculture Setup

**Day 0 – Endothelial Seeding**: EECM‐BMECs (2 × 10⁶ cells mL^−1^) were seeded into the flow channel of microfluidic µSiM devices precoated with collagen IV/fibronectin. A 75 µL droplet of hECSR was layered atop PDMS inserts to minimize evaporation, with daily media replenishment. **Day 3 – Astrocyte Seeding**: PHAs (33 000 cells cm^−^^2^) were seeded on the floor of the bottom channel, precoated with collagen I/fibronectin to mimic the anatomical arrangement of endothelial–astrocyte interactions in postcapillary venules—the most reactive sites to inflammation and major sites of leukocyte trafficking.^[^ ^28^ ^]^ PHAs were maintained in AGM, while EECM‐BMECs remained in hECSR with 75 µL droplet atop a flow insert. **Day 4—Flow Initiation**: Laminar flow (0.5 Pa shear stress) was introduced to the matured EECM‐BMEC barrier in the fluidic groups using the customized flow circuit mentioned above, while static groups were maintained without flow. Fluidic control groups sustained a stable 0.5 Pa shear stress for 48 h. **Day 5—Inflammatory Challenge**: For inflammatory stimulation, fluidic groups were exposed to inflammatory stimuli by replacing circulating media with 5 mL of one of the following treatments (prepared in hECSR): 50 pg mL^−1^ cytomix alone, 2.5 mg 50 pg mL^−1^ fibrinogen alone, or a combination of 50 pg mL^−1^ cytomix and 2.5 mg mL^−1^ fibrinogen together. Treatments were circulated continuously (0.5 Pa shear stress) for 24 h. **Day 6—Endpoint Analyses**: Experiments were terminated for in situ barrier permeability measurement using 457 Da lucifer yellow and astrogliosis quantification via GFAP immunocytochemistry.

### Cell Alignment Analysis

Phase‐contrast live‐cell imaging was performed using an inverted microscope (Olympus CK2, 4× or 10× objective) equipped with a Canon EOS T3i camera to capture cellular morphology under shear stress without fixation, ensuring cell viability for subsequent in situ permeability assays. Cell orientation angles relative to the flow direction (90°) were quantified using the Analyze Directionality function in FIJI/ImageJ. Directionality histograms (0–180° bins, 10° intervals) were generated in GraphPad Prism 10 (v10.4.1). Cells were considered aligned if their orientation angles fell within ± 30° of the flow direction (60°–120°). Total cell numbers were quantified using the Analyze Particles function in FIJI/ImageJ, ensuring objective and automated cell identification for alignment percentage calculations. Alignment percentages were calculated as:

(1) $$\%\;\mathrm{Aligned}\;\mathrm{Cells}=\frac{\mathrm{Number}\;\mathrm{of}\;\mathrm{aligned}\;\mathrm{cells}\;\left(60^{\circ }-120^{\circ }\right)}{\mathrm{Total}\;\mathrm{number}\;\mathrm{of}\;\mathrm{analyzed}\;\mathrm{cells}\;}\times 100$$

### In Situ Permeability Measurements

Endothelial barrier permeability was quantified using an adapted confocal microscopy protocol^[^ ^8^ ^]^ with modifications to optimize compatibility with the µSiM fluidic platform (Figure S2, Supporting Information). Briefly, a fluorescent small‐molecule tracer (lucifer yellow, Thermo Fisher, 150 µg mL^−1^ in hECSR) was introduced into the luminal (“blood”) flow channel and layered 75 µL atop the PDMS insert, different from the prior methods where dye was added to the open‐well. This adjustment was necessitated by the flow insert's occupation of the open well, which reduced the flow channel volume to 1.22 µL, insufficient to maintain a constant dye concentration gradient across the endothelial barrier. Permeability assays were conducted using an Andor Spinning Disk Confocal microscope (10× objective, NA 0.45) with the focal plane positioned 100 µm below the nanomembrane surface to capture tracer diffusion into the abluminal (“brain”) compartment. Time‐lapse imaging (1 frame min^−1^ for 10 min) was initiated immediately after tracer introduction. Permeability (*P*) was calculated using the Constant Flux equation:

(2) $$\frac{F_{x,t}-F_{b}}{F_{0}-F_{b}}=\left(2\sqrt{\frac{t}{\pi D}}\;exp\left(-\frac{x^{2}}{4\mathrm{Dt}}\right)-\frac{x}{D}erfc\left(\frac{x}{2\sqrt{Dt}}\right)\right)P$$

where ***F_x_*_,_ *_t_*** is fluorescence intensity at position ***x*** and time ***t***, ***F_b_*** is background intensity, ***F*_0_** is source intensity, ***D*** is the tracer's diffusion coefficient, and ***x*** is diffusion distance. Diffusion coefficients were derived via the Stokes–Einstein equation at 20 °C (water viscosity = 1.002 mPa·s).

### COMSOL Multiphysics—Shear Stress Profiling

The shear stress profile over the endothelial culture area was modeled using COMSOL Multiphysics (v6.1, Laminar Flow Module), as previously validated.^[^ ^13^, ^25^ ^]^ Briefly, Laminar Flow module simulated fluid dynamics within the flow channel, incorporating: 1) boundary conditions—no‐slip at walls, inlet flow rate = 290 µL min^−1^ (achieving 0.5 Pa shear stress), and outlet pressure = 0 Pa; 2) mesh—physics‐controlled tetrahedral mesh (minimum element size = 10 µm). The model confirmed uniform shear stress across the membrane culture area (Figure 1B).

### COMSOL Multiphysics—COMSOL Validation of Permeability Methodology

A COMSOL Multiphysics model (v6.1) was developed (Figure S7, Supporting Information) to validate the modified permeability assay for fluidic µSiM‐BBB (Figure S2, Supporting Information). The simulation replicated the experimental geometry, including the luminal (“donor”) and abluminal (“receiver”) compartments separated by a 5 µm‐thick endothelial layer with a membrane layer. Tracer dynamics were modeled for 75 µL of Lucifer Yellow (150 µg mL^−1^).

### Secreted CXCL8 Quantification

Secreted CXCL8 levels were quantified from basal (abluminal/brain) compartments of EECM‐BMEC monocultures using a Human IL‐8/CXCL8 ELISA Kit (R&D Systems, D8000C). Samples were collected from four experimental groups: static control, fluidic control (0.5 Pa, 48 h), static inflammatory (50 pg mL^−1^ cytomix), and fluidic inflammatory (50 pg mL^−1^ cytomix + 0.5 Pa). For basal compartment sampling, 200 µL of hECSR was introduced into one port of the bottom channel to establish a reservoir, and the total volume (including the native 10 µL channel volume) was collected via reverse pipetting from the opposite port. All samples were centrifuged (300 × g, 5 min) to pellet debris and stored at −80 °C prior to analysis.

### Immunofluorescence and Confocal Imaging

Immunostaining protocols were performed as follows, with variations in antibodies and conditions tailored to each target (ICAM‐1, HA, or GFAP). For all procedures, cells were fixed with 4% paraformaldehyde (PFA, Invitrogen) for 10 min at room temperature (RT), washed three times with 1× PBS (Gibco), and blocked with 5% goat serum (Invitrogen) containing 0.1% Triton X‐100 for 30 min at RT. Nuclei were counterstained with Hoechst 33342 (Molecular Probes, 1:1000), and images were acquired using an Andor Spinning Disk Confocal microscope with 10× (NA 0.45), 20× (NA 0.75), or 40× (NA 1.15) objectives. ICAM‐1: Live EECM‐BMECs were incubated with anti‐human CD54 primary antibody (HA58, BioLegend, 1:100 in hECSR) for 15 min at 37 °C/5% CO_2_ prior to fixation. After fixation and blocking, cells were incubated with goat anti‐mouse IgG Alexa Fluor 488 (A11011, Life Technologies, 1:200) for 1 h at RT. HA: Fixed cells were incubated with biotinylated HA‐binding protein (Sigma–Aldrich, 385911, 1:100) for 1 h at RT, followed by three luminal washes and one abluminal wash. Biotin‐conjugated secondary antibodies (Alexa Fluor 546, sc‐101339, Santa Cruz, 1:200) were applied for 1 h. GFAP: PHAs were incubated with GFAP primary antibodies (rabbit monoclonal EP672Y, Abcam, or mouse monoclonal ASTRO6, Invitrogen, 1:100) for 1 h at RT. Species‐matched secondary antibodies (goat anti‐rabbit IgG Alexa Fluor 568, A11011, or goat anti‐mouse IgG Alexa Fluor 488, A11001, Life Technologies, 1:200) were used for detection. All images were processed using Imaris (v10.2) with consistent thresholding and background subtraction across experimental groups.

### Image Analysis—Glycocalyx (HA) Measurement

HA distribution within the endothelial glycocalyx was quantified by analyzing the MFI of Z‐projected confocal stacks (average intensity) using Fiji/ImageJ. Z‐stacks of EECM‐BMECs stained for HA were acquired under identical imaging parameters (laser power, exposure, gain) to enable cross‐condition comparisons.

### Image Analysis—ICAM‐1 Expression Quantification

ICAM‐1 MFI was quantified across Z‐stack images using Fiji/ImageJ's maximum intensity projection to assess total protein expression within endothelial monolayers. Z‐stacks of EECM‐BMECs stained for ICAM‐1 were acquired under identical imaging parameters (laser power, exposure, gain) to enable cross‐condition comparisons.

### Image Analysis—Astrocyte Morphometric Analysis

Astrocyte branching complexity was quantified via skeleton analysis of GFAP^+^ PHAs using Fiji/ImageJ.^[^ ^86^ ^]^ Z‐stacks (≥ 30 optical slices at 1 µm intervals) were acquired from moderate‐density regions in which more than 80% astrocytes were morphologically distinguishable. Maximum intensity projections were exclusively used to accurately capture 3D branching morphology, minimizing the possibility that apparent elongation or branching complexity results from planar distortion or partial visualization. Only astrocytes with clearly traceable somas with minimal processes overlap were analyzed to prevent misinterpretation of interconnected networks. Imaging was performed under standardized parameters (laser power, exposure, gain) to ensure cross‐condition comparability and to capture complete 3D astrocytic morphology independent of imaging plane. The detailed workflow (shown in Figure S8, Supporting Information) included: preprocessing, 3D skeletonization with soma‐anchored tracing, and quantification of branch length and branch number per 100 cells. Total cell numbers were determined using the Analyze Particles function in FIJI/ImageJ, ensuring objective and automated cell identification for alignment percentage calculations. The number of branches per 100 cells was quantified by counting cells with more than three branches. Data were pooled across ≥ 3 biological replicates (independent astrocyte cultures) and analyzed using GraphPad Prism 10.0

### Neutrophil Transmigration

Neutrophils were isolated from heparinized whole blood obtained from healthy donors. Written informed consent was obtained from all participants prior to blood collection in accordance with protocols approved by the University of Rochester Institutional Review Board (study/project number STUDY0004777). Ethical approval for this study was granted by the same institutional review board. A standardized density gradient centrifugation protocol^[^ ^56^, ^77^, ^87^ ^]^ was employed: whole blood was combined with an equal volume of 1‐Step Polymorphs solution (Accurate Chemical & Scientific Co.), centrifuged (500 × g for 30 min at 20 °C), and the polymorphonuclear (PMN) layer was harvested. Residual erythrocytes were lysed using hypotonic PBS (1:6 dilution, 4.5 mL, 1 min), followed by isotonic restoration (1.5 mL of 4× PBS) and centrifugation (350 × g, 10 min). Purified neutrophils were resuspended in neutrophil isolation buffer (HBSS without Ca^2^⁺/Mg^2^⁺, 10 mM HEPES, 5 mg mL^−1^ BSA) and maintained under gentle rotation to prevent aggregation.

For confocal tracking, neutrophils were labeled with 5 µM DiD Vybrant Multicolor Cell‐Labeling Solution (Invitrogen, V22889) for 20 min at 37 °C, followed by three washes with serum‐free medium via centrifugation (350 × g, 5 min).^[^ ^9^ ^]^ Unlabeled neutrophils were used for brightfield imaging.

For tthe he neutrophil transmigration assay, EECM‐BMECs were cultured in µSiMs with 0.625% porosity, 3 µm dual‐scale membranes. Prior to experiments, luminal channels were treated with either hECSR (control) or cytomix‐supplemented hECSR (inflammatory groups). Physiological concentrations of neutrophils (3 × 10⁶ cells mL^−1^ in hECSR^)[^ ^84^ ^]^ were introduced into the luminal (flow) channel via pipette injection, followed by a 75 µL droplet of corresponding media atop the PDMS insert. After 1.5‐h incubation, non‐adherent cells were removed by gentle rinse (10 µL hECSR ×2). Neutrophil adhesion and transmigration were quantified: 1) DiD‐Labeled Cells: Confocal Z‐stacks (2 µm intervals; Andor Spinning Disk, 10× objective) were acquired to differentiate luminal adhesion (DiD⁺ cells adherent to endothelium) from abluminal transmigration (DiD⁺ cells in the basolateral compartment).^[^ ^25^ ^]^ 2) Unlabeled Cells: Brightfield imaging (Andor Spinning Disk, 10× objective) enabled morphology‐based quantification, which validated consistency with DiD‐labeled results (Figure S9, Supporting Information).

FIJI/ImageJ software was used to quantify neutrophils via the Analyze Particles function. A color threshold (DiD⁺) or size/shape threshold (brightfield) was applied to distinguish neutrophils on the EECM‐BMEC barrier (luminal adhesion) or the floor of the abluminal channel (transmigrated). Total attached neutrophils were calculated as the sum of transmigrated and luminal‐adherent populations.

(3) $$\%\;\mathrm{Transmigrated}=\frac{\mathrm{Number}\;\mathrm{of}\;\mathrm{transmigrated}\;\mathrm{neutrophils}}{\mathrm{Total}\;\mathrm{neutrophils}\;\mathrm{added}\;}\times 100$$

(4) $$\begin{array}{c} \%\;\mathrm{Total}\;\mathrm{adhered}=\frac{\mathrm{Transmigrated}+\mathrm{Luminal}-\mathrm{adhered}\;\mathrm{neutrophils}\;}{\mathrm{Total}\;\mathrm{neutrophils}\;\mathrm{added}\;}\times 100 \end{array}$$

### Statistical Analysis

Data are reported as mean ± standard deviation (SD) unless otherwise specified. For parametric comparisons across ≥ 3 groups, one‐way ANOVA with Tukey's post hoc test was applied. All experiments included ≥ 3 technical replicates per condition to ensure reproducibility. Statistical significance was defined as: ∗∗∗∗*p* < 0.0001, ∗∗∗*p* < 0.001, ∗∗*p* < 0.01, ∗∗*p* < 0.05, ns > 0.05. Analyses were performed using GraphPad Prism 10.

## Conflict of Interest

J.L.M. is a cofounder of SiMPore and holds an equity interest in the company. SiMPore is commercializing ultra‐thin silicon‐based technologies, including the membranes used in this study. B.E. is an inventor on a provisional U.S. patent application (63/185815) related to the methodology of EECM‐BMEC‐like cell differentiation.

## Author Contributions

K.C. performed conceptualization, investigation, visualization, validation, project administration, and formal analysis, developed the methodology, worked with the software, curated data, wrote the original draft, and acquired funds. J.L.M. performed conceptualization and supervision, developed the methodology, acquired funds and resources, and wrote, reviewed, and edited the final draft. I.M.L. developed the methodology and performed the investigation. M.A.T. and P.K. performed the investigation (Differentiation and Maintenance of EECM‐BMECs) and acquired resources. A.M.F. developed the methodology and performed the investigation. D.D.S. and S.F. performed validation and worked with the software (COMSOL Validation of Tracer Retention). J.J. performed the investigation (Performed De‐Alignment Assay at the 6‐h Timepoint). J.K. and R.E.W. performed conceptualization (HA Expression Assays) and developed the methodology. H.A.G. and N.T. performed conceptualization (Combined Inflammatory Insults) and developed the methodology. B.E., J.F., V.V.A., R.E.W., H.A.G., and N.T. wrote, reviewed, and edited the final draft. All authors reviewed and approved the final manuscript.

## Supporting information



Supporting Information

## Data Availability

The data that support the findings of this study are available from the corresponding author upon reasonable request.
