# Supplementary material for: Shear Conditioning Promotes Microvascular Endothelial Barrier Resilience in a Human BBB‐on‐a‐Chip Model of Systemic Inflammation Leading to Astrogliosis
Source: Adv Sci (Weinh). 2025 Aug 27;12(43):e08271. doi: 10.1002/advs.202508271 (PMC12631881; doi:10.1002/advs.202508271)
Supplement: Supplementary file 1 — Supporting Information [file ADVS-12-e08271-s001.docx]

**Shear Conditioning Promotes Microvascular Endothelial Barrier Resilience in a Human BBB-on-a-Chip Model of Systemic Inflammation Leading to Astrogliosis**

*Authors:* *Kaihua Chen, Isabelle M. Linares, Michelle A. Trempel, Alexis M. Feidler, Dinindu De Silva, Sami Farajollahi, Jordan Jones, Julia Kuebel, Pelin Kasap, Britta Engelhardt, Jonathan Flax, Vinay V. Abhyankar, Richard E. Waugh, Harris A. Gelbard, Niccolo Terrando, James L. McGrath**

K. Chen^1^, I. M. Linares^1,2^, M. A. Trempel^1^, A. M. Feidler^3^, D. De Silva^4^, S. Farajollahi^4^, J. Jones^1^, J. Kuebel^1^, P. Kasap^5^, B. Engelhardt^5^, J. Flax,^1,6^ V. V. Abhyankar^4^, R. E. Waugh^1^, H. A. Gelbard^7,8,9,10^, N. Terrando^11,12,13^, J. L. McGrath^1*^

^1^ = Department of Biomedical Engineering, University of Rochester, Rochester, NY, United States.

^2^ = Center for Musculoskeletal Research, University of Rochester Medical Center, Rochester, NY, United States.

^3^ = Department of Neuroscience, University of Rochester, Rochester, NY, United States.

^4^ = Department of Biomedical Engineering, Rochester Institute of Technology, Rochester, NY, United States.

^5^ = Theodor Kocher Institute, University of Bern, Bern, Switzerland

^6^ = Department of Urology, University of Rochester Medical Center, Rochester, NY, United States.

^7^ = Center for Neurotherapeutics Discovery, Department of Neurology, University of Rochester Medical Center, Rochester, NY, United States

^8^ = Department of Neurology, University of Rochester Medical Center, Rochester, NY, United States

^9^ = Department of Neuroscience, University of Rochester Medical Center, Rochester, NY, United States

^10^ = Department of Microbiology and Immunology, University of Rochester Medical Center, Rochester, NY, United States

^11^ = Department of Anesthesiology, Center for Translational Pain Medicine, Duke University Medical Center, Durham, NC, United States.

^12^ = Department of Cell Biology, Duke University Medical Center, Durham, NC, United States.

^13^ = Department of Immunology, Duke University Medical Center, Durham, NC, United States.

*Corresponding Author: jmcgrath@bme.rochester.edu, 585-273-5489

**Key words:** fluid shear stress, human BBB-on-a-chip, barrier resilience, astrogliosis

**Supplementary Materials**

**Figure S1**. **Shear Stress (SS) Accelerates EECM-BMEC Alignment in a Time- and Magnitude-Dependent Manner.** EECM-BMECs exposed to 0.5 Pa SS achieved significant alignment (74.20 ± 6.74% for 48 h, 72.95 ± 6.64 for 72 h, one way ANOVA with Tukey’s post-hoc, ∗∗∗∗p < 0.0001 vs. static controls 29.05 ± 2.08%) within 48 hours, compared to prolonged 72-hour treatments under lower SS conditions (67.08 ± 7.45% for 0.1 Pa, 70.37 ± 4.05 for 0.25 Pa). No significant differences were observed between 48-hour and 72-hour cohorts (ns > 0.05, student t test) under 0.5 Pa SS, or between groups after 72 hours across all SS magnitudes (ns > 0.05, ANOVA with Tukey’s post hoc). These findings demonstrate that 0.5 Pa SS applied for 48 hours is sufficient to achieve maximal alignment, establishing an optimized protocol for investigating SS-driven mechanotransduction in engineered neurovascular models. n = 4–7 for each condition.

**Figure S2**. **Workflow for In Situ Permeability Measurements in the Fluidic µSiM-BBB Model.** Schematic of the non-destructive lucifer yellow permeability assay designed for assessing barrier integrity post physiological shear. Stepwise procedure: (1) Flow arrest: Media (50 µL) is added to the apical insert to prevent evaporation in the luminal channel during flow cessation. (2) Pressure stabilization: Tubing occlusion via clamps maintains hydrostatic equilibrium in the flow circuit. (3) Device isolation: Inlet/outlet needles are removed to decouple the µSiM-BBB from the peristaltic pump. (4) Microscopy mounting: The device is mounted on a confocal microscope stage (Andor Spinning Disc Confocal Microscope) for time-lapse imaging. (5) Tracer introduction: Luminal introduction of lucifer yellow (457 Da, 150 µg mL⁻¹)—10 µL initial bolus followed by 75 µL continuous infusion—ensures a stable tracer gradient. (6) Real-time quantification: Fluorescence intensity in the abluminal compartment is imaged at *t* = 0, 1, 6, and 10 min to calculate apparent permeability coefficients using **Equation** **(2)**.

**
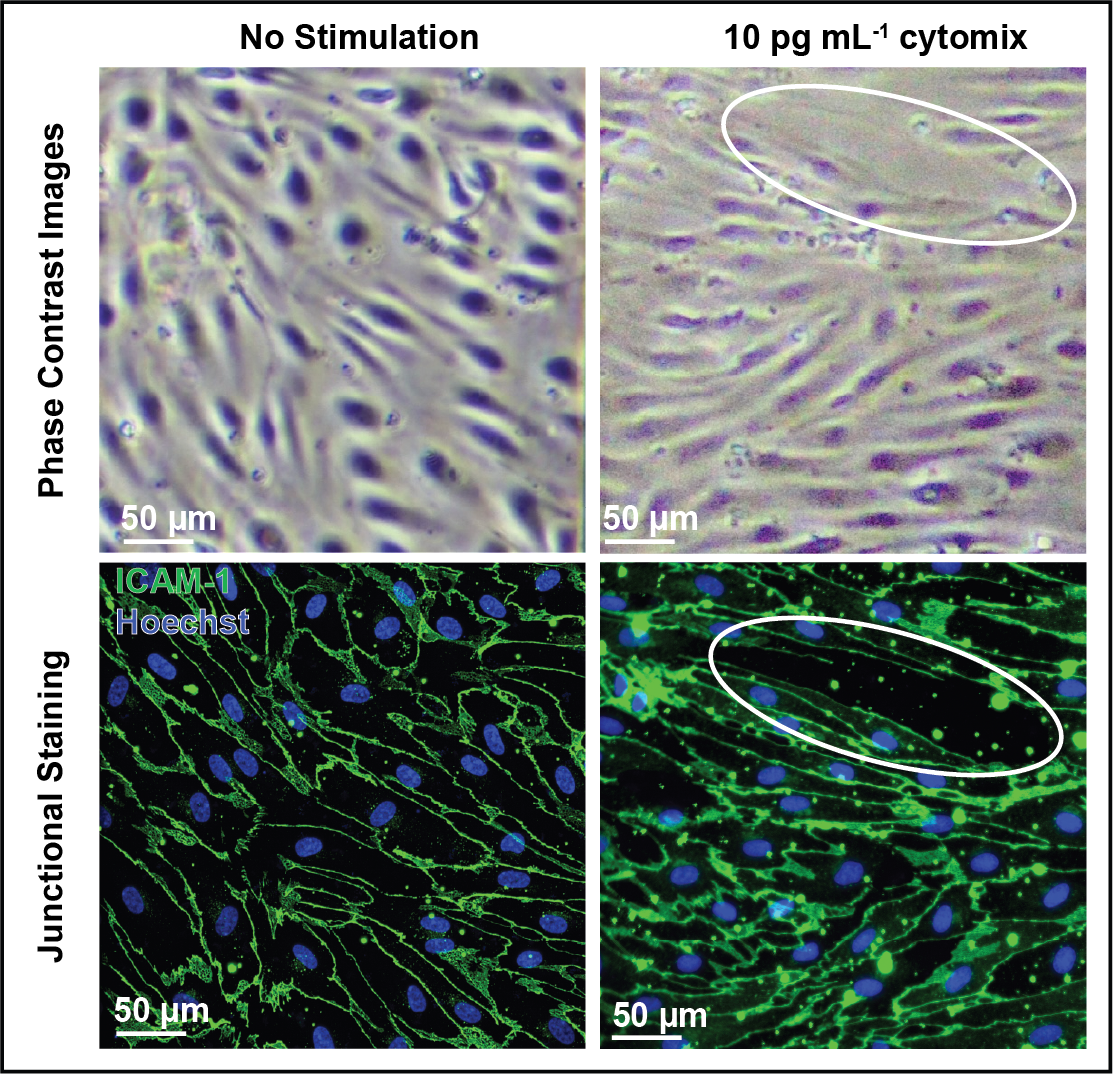
**

**Figure S3**. **Multimodal Validation of Endothelial Barrier Disruption under Cytomix (10 pg mL⁻¹).** Representative phase-contrast images (top) and corresponding VE-cadherin immunofluorescence (bottom) of EECM-BMEC monolayers with (left) and without (right) static 10 pg mL⁻¹ of cytomix exposure. White ovals highlight barrier gaps defined as: > 20 μm discontinuities lacking nuclei and VE-cadherin signal, confirming structural compromise. Scale bars = 50 μm. VE-cadherin stained with mouse anti-human CD144 antibody (R&D Systems MAB9381, 1:100; Alexa Fluor 488 secondary). Imaging: Andor Spinning Disk Confocal (40× water).


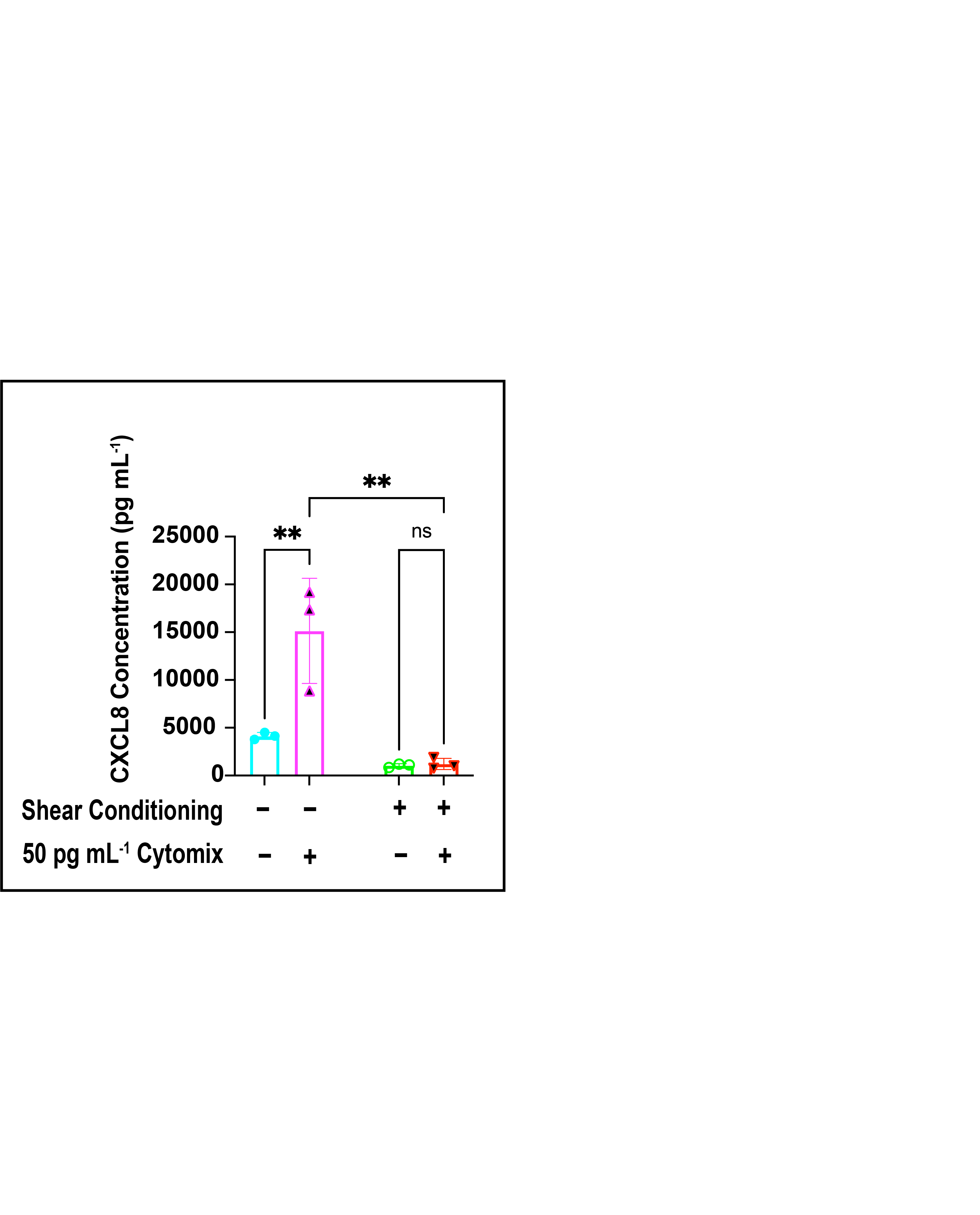


**Figure S4. Shear Conditioning Ablates Pathological Basal CXCL8 Gradients.** CXCL8 secretion in EECM-BMECs after 24 h exposure to 50 pg mL⁻¹ cytomix under static vs. shear-preconditioned (0.5 Pa, 48 hr) conditions. Static barriers exhibited pathological 3.7-fold increase in basal (brain-side) CXCL8 (∗∗*p* < 0.01), whereas shear-conditioned endothelia showed no increase (*p* > 0.05). As basal CXCL8 levels have been established as the primary driver of neutrophil transmigration across endothelial monolayers^[1, 2]^, explaining absent diapedesis in shear groups (**Figure 7B**). Data: mean ± SD; n=4; one-way ANOVA, Tukey’s post-hoc.

**Figure S5**. **Barrier Disruption in the Fluidic µSiM-BBB with EECM-BMEC/PHA Co-Cultures.** Representative immunofluorescence images of ZO-1 expression (green) in fluidic EECM-BMEC/PHA co-cultures treated with 50 pg mL⁻¹ cytomix alone, 2.5 mg mL⁻¹ fibrinogen alone, or a combination of 50 pg mL⁻¹ cytomix + 2.5 mg mL⁻¹ fibrinogen. Endothelial barriers were greatly disrupted in response to 50 pg mL^-1^cytomix, or combined 50 pg mL^-1^cytomix + 2.5 mg mL^-1^ fibrinogen, demonstrating endothelial dysfunction after high-dose inflammatory cytokines exposure. Gaps are defined as discontinuities > 20 μm in the endothelial monolayer, characterized by the loss of cell–cell contact and absence of nuclei within the void area. Hoechst nuclear stain (blue) marks cell nuclei. White oval circles highlight barrier gaps post-cytomix treatment. Scale bar = 50 μm.

**Figure S6. Absence of Astrocyte Activation in PHA Monocultures Exposed to Combined 50 pg mL⁻¹ Cytomix and 2.5 mg mL⁻¹ Fibrinogen Highlighting Endothelial-Dependent Neuroinflammatory Amplification.** Representative immunofluorescence images of GFAP (green) and Hoechst (blue) in PHAs cultured under control conditions, 50 pg mL⁻¹ cytomix alone, 2.5 mg mL⁻¹ fibrinogen alone, and combined cytomix + fibrinogen for 24 hours, indicating that astrocyte activation in cocultures (**Figure 9**) requires endothelial-dependent mechanisms. Scale bar = 100 µm.

**Figure S7**. **Computational Validation of Tracer Retention in the µSiM-BBB Donor Compartment.** (A) Time-lapse simulation of Lucifer Yellow (150 µg mL⁻¹, 75 µL) diffusion across a total of 5 µm-thick endothelial layer in the µSiM-BBB. Snapshots show tracer distribution in the luminal ("donor") compartment at t = 0, 6, and 10 min. Simulations performed using COMSOL Multiphysics with parameters matching experimental conditions (see Methods). **(B)** Time-dependent mass transfer to the abluminal ("receiver") compartment, calculated as M_trench_(*t*) / M_initial_ × 100%, where M_trench_ is the integrated tracer mass in the receiver compartment and M_initial_​ is the initial tracer mass in the donor compartment. Dashed line indicates that the transfer in 10 minutes stays below the 10% receiver compartment threshold for valid Transwell assays.^[3]^

**Figure S8**. **Skeleton analysis of GFAP^+^ PHAs.**^[4]^ Preprocessing: Binary conversion of GFAP channel images using a fixed intensity threshold to isolate astrocyte processes. Skeletonization: Application of the Skeletonize 3D plugin to reduce processes to 1-pixel-wide skeletons, preserving branch topology. Quantification: Automated measurement of total branch length and branchpoint counts per cell using the Analyze Skeleton (2D/3D) function.

**Figure S9**. **No Significant Difference of Adhesion and Transmigration between DiD-Labeled and Unlabeled Neutrophils.** Comparison of neutrophil adhesion and transmigration between unlabeled (blue) and Vybrant™ DiD-labeled (cyan) neutrophils across four experimental conditions: Static Control: Non-stimulated baseline. Static Inflammatory: Cytomix exposure (10 pg mL⁻¹ TNF-α/IL-1β/IFN-γ). Shear-Conditioned (48 h): Physiological shear (0.5 Pa). Pre-Shear (24 h) + Circulating Cytokine (24 h): Sequential shear conditioning followed by cytomix perfusion.

**
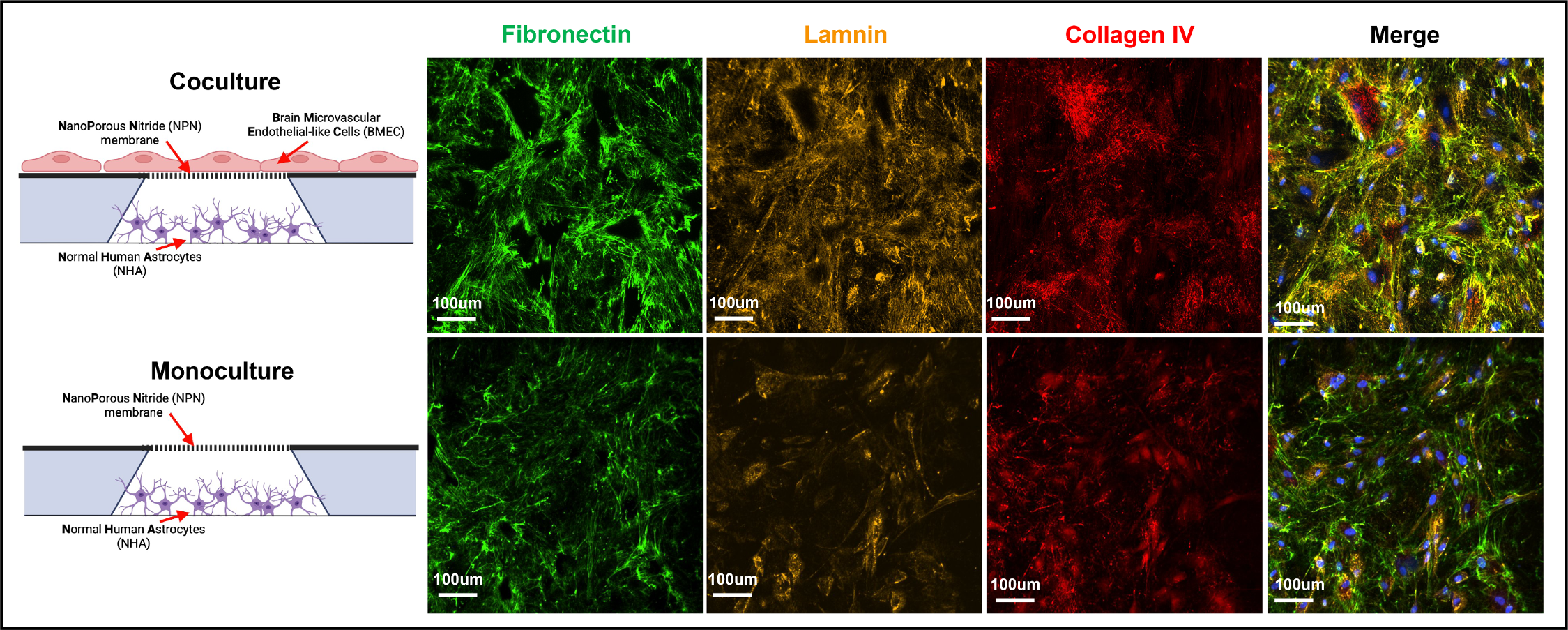
**

**Figure S10. Astrocyte-Generated Basement Membrane Remodeling in Coculture.** Representative confocal images comparing basement membrane (BM) protein deposition by primary human astrocytes (PHAs/NHAs) in coculture with EECM-BMECs (top) versus monoculture (bottom). Samples were immunostained for Hoechst (nuclei, blue), Fibronectin (green), Laminin (orange), and Collagen IV (red). Scale bars: 100 µm. To investigate the astrocytic contributions to barrier integrity, we analyzed basement membrane (BM) protein expression (laminin, collagen IV, fibronectin) in PHA monocultures versus EECM-BMEC/PHA cocultures. Astrocytes secrete BM proteins in distinct patterns depending on culture context. In coculture, PHAs exhibit polarized, fibrous deposition of Laminin and Collagen IV, reminiscent of in vivo glia limitans maturation, while monocultures show more punctate, pericellular localization centered around cell bodies. This structural reorganization did not enhance barrier function (fluidic coculture permeability: 0.77 ± 0.12 × 10⁻³ cm min⁻¹ vs. EECM-BMEC monoculture: Pe*_LY_*  < 0.6 × 10^-3^ cm min^-1^; **Figure 4A**), likely attributable to the 310 µm bottom channel separation between astrocytes and endothelia, which physically uncouples BM deposition from endothelial junctional complexes. Fibronectin distribution appeared similar between groups. **Experimental timeline:** On Day 0, EECM-BMECs (40,000 cells cm⁻²) were seeded into collagen IV/fibronectin-coated (400/100 µg mL⁻¹) luminal channels. On Day 3, PHAs (33,000 cells cm⁻²) were seeded into collagen I/fibronectin-coated (100/50 µg mL⁻¹) abluminal channels to establish cocultures. PHA monocultures followed the same coating and seeding timeline but did not include endothelial cells. **Immunostaining protocol:** On Day 6, devices were live-stained for Collagen IV (Invitrogen 51-9871-82), Fibronectin (Invitrogen 53-9869-82), and Laminin (Invitrogen PA1-16730) for 2 hours, followed by PBS washes and fixation with 4% paraformaldehyde (15 min). Samples were then blocked with 10% normal goat serum (10 min), incubated with secondary antibodies (if needed), and counterstained with Hoechst. Imaging was performed using a Dragonfly confocal microscope. Results reflect n = 4 for coculture and n = 3 for monoculture.

**Table S1**. **Total PHA cell counts per device replicate used for branch quantification (branches per 100 cells) in** **Figure 8**.

|  |  |  |  |  |  |
| --- | --- | --- | --- | --- | --- |
|  | **Non-Stim** | **100 pg mL^-1^** | **1 ng mL^-1^** | **10 ng mL^-1^** | **100 ng mL^-1^** |
|  | 356 | 313 | 310 | 415 | 462 |
|  | 311 | 333 | 462 | 677 | 677 |
|  | 355 | 275 | 706 | 361 | 401 |
|  | 309 | 379 | 469 | 311 | 441 |
|  | 370 | 343 | 397 | 501 | 392 |
|  | 294 |  | 342 | 451 |  |
|  | 360 |  | 333 | 363 |  |
|  | 373 |  | 264 |  |  |
|  | 281 |  | 255 |  |  |
|  |  |  |  |  |  |

**References**

1. Salminen, A. T.; Tithof, J.; Izhiman, Y.; Masters, E. A.; McCloskey, M. C.; Gaborski, T. R.; Kelley, D. H.; Pietropaoli, A. P.; Waugh, R. E.; McGrath, J. L., *Integr Biol (Camb)* **2020,** *12* (11), 275-289. DOI 10.1093/intbio/zyaa022.

2. Ahmad, D.; Linares, I.; Pietropaoli, A.; Waugh, R. E.; McGrath, J. L., *Adv Healthc Mater* **2024,** *13* (21), e2304338. DOI 10.1002/adhm.202304338.

3. Hubatsch, I.; Ragnarsson, E. G. E.; Artursson, P., *Nature Protocols* **2007,** *2* (9), 2111-2119. DOI 10.1038/nprot.2007.303.

4. Arganda-Carreras, I. <https://imagej.net/plugins/skeletonize3d>.
